# Regular human insulins versus rapid-acting insulin analogues in children and adolescents with type 1 diabetes: a systematic review with meta-analysis and trial sequential analysis

**DOI:** 10.1136/bmjopen-2025-100186

**Published:** 2026-05-14

**Authors:** Johanne Juul Petersen, Pascal Faltermeier, Sophie Juul, Caroline Barkholt Kamp, Christina Dam Bjerregaard Sillassen, Tiago Jeronimo Dos Santos, Janus Christian Jakobsen

**Affiliations:** 1 Copenhagen Trial Unit, Centre for Clinical Intervention Research, Rigshospitalet, Copenhagen, Denmark; 2 Faculty of Health and Medical Sciences, University of Copenhagen, Copenhagen, Denmark; 3 Mental Health Services in the Capital Region of Denmark, Psykoterapeutisk Center Stolpegard, Gentofte, Denmark; 4 Department of Psychology, University of Copenhagen, Copenhagen, Denmark; 5 Department of Regional Health Research, Faculty of Health Sciences, University of Southern Denmark, Odense, Denmark; 6 Department of Cardiology, Central and West Zealand Hospital, Slagelse, Denmark; 7 Unit of Pediatrics, Hospital Vithas Almería, Instituto Hispalense de Pediatría SL, Seville, Spain; 8 Department of Nursing Physiotherapy and Medicine, Faculty of Health Sciences, University of Almeria, Almería, Spain

**Keywords:** DIABETES & ENDOCRINOLOGY, Paediatric endocrinology, Systematic Review, Meta-Analysis

## Abstract

**Abstract:**

**Objectives:**

To assess the beneficial and harmful effects of regular human insulins versus rapid-acting insulin analogues in children and adolescents with type 1 diabetes.

**Design:**

Systematic review of randomised clinical trials with meta-analysis and trial sequential analysis.

**Data sources:**

CENTRAL, MEDLINE, Embase, LILACS and other sources from inception to 30 January 2026.

**Study selection:**

Randomised clinical trials comparing regular human insulins versus rapid-acting insulin analogues (insulin aspart, lispro, glulisine) in children and adolescents with type 1 diabetes.

**Analyses:**

Data were analysed using meta-analysis and trial sequential analysis. Risk of bias was assessed using the Cochrane Risk of Bias tool, V.2, and the certainty of the evidence was assessed using the Grading of Recommendations Assessment, Development and Evaluation (GRADE) approach.

**Primary outcomes:**

Severe hypoglycaemia, ketoacidosis and serious adverse events.

**Results:**

10 trials randomising 1107 participants were included. The certainty of evidence was very low mainly due to high risk of bias and small sample sizes. Meta-analysis showed no evidence of a difference between regular human insulins and rapid-acting insulin analogues on severe hypoglycaemia (risk ratio (RR) 1.28, 95% CI 0.81 to 2.03; I^2^=0.0%; p=0.2851; nine trials), ketoacidosis (RR 0.88, 95% CI 0.26 to 2.93; I^2^=0.0%; p=0.8593; two trials) and serious adverse events (RR 1.00, 95% CI 0.44 to 2.25; I^2^=0.0%; p=0.9958; two trials). Trial sequential analysis showed that all meta-analyses of primary outcomes were underpowered.

**Conclusions:**

Current research shows no differential effects between regular human insulins and rapid-acting insulin analogues for children and adolescents with type 1 diabetes, but the evidence is very uncertain.

**PROSPERO registration number:**

CRD42024508625.

**STRENGTHS AND LIMITATIONS OF THIS STUDY**

- This review was conducted in accordance with a predefined methodology outlined in both a published protocol and registry.
- Both the risks of random and systematic errors were considered in this review by using risk of bias assessments with the Cochrane Risk of Bias tool V.2 and trial sequential analysis.
- The results of this review may be susceptible to publication bias, because only a limited amount of grey literature was identified.
- Since the review is based on data from crossover trials, it is not possible to draw conclusions about long-term effects, and the relevance of short-term effects may be limited due to the potential risk of carryover effects.

## Introduction

 Type 1 diabetes is a chronic health condition with a rising incidence.^1 2^ The disease is characterised by autoimmune destruction of pancreatic β-cells resulting in insulin deficiency and hyperglycaemia.^3 4^ Type 1 diabetes is associated with short-term complications (such as ketoacidosis) and long-term complications (such as microvascular and macrovascular diseases).^5^

Pharmacological interventions often consist of basal insulin combined with a mealtime bolus.^6 7^ The mealtime bolus is available as regular human insulins or rapid-acting insulin analogues (insulin aspart, lispro or glulisine).^8 9^ Although previous guidelines in paediatric population highlight that rapid-acting insulin analogues can offer superior postprandial glucose control and reduce the risk of hypoglycaemia, especially nocturnal hypoglycaemia, the selection of insulin type should be individualised to meet the specific needs and lifestyle of the paediatric patient, making it important to assess both types of insulin in this population to determine their comparative benefits and risks.^10 11^ Additionally, administration of insulin may lead to additional complications (such as hypoglycaemia).^5 12^

Previous reviews have focused on adults with type 1 diabetes.^13^,^16^ Children and adolescents differ from the adult population on several parameters including metabolic control, risk of hypoglycaemia, developmental considerations and involvement of parents/caregivers.^17^ A previous 2019 review compared regular human insulin with rapid-acting insulin analogues in children, adolescents and adults.^18^ However, only five randomised clinical trials with children and adolescents were included.^18^ Another review from 2018 included eight randomised clinical trials with children and adolescents.^19^ However, it was limited by not publishing a protocol in advance, not searching all relevant databases, not employing trial sequential analysis methods to control for random error, not assessing risk of bias and not assessing the certainty of evidence using Grading of Recommendations Assessment, Development and Evaluation (GRADE).^19^ Thus, the objective of this systematic review was to assess the beneficial and harmful effects of regular human insulins versus rapid-acting insulin analogues in children and adolescents with type 1 diabetes, that is, in children and adolescents with type 1 diabetes what type of insulin is the optimal treatment?

## Methods

This systematic review is reported based on the Preferred Reporting Items for Systematic Reviews and Meta-Analysis (PRISMA)^20^ guidelines (online supplemental text S1).^21^ The updated methodology was conducted according to our protocol,^13^ registered in the PROSPERO database (ID: CRD42024508625) before our literature searches.

### Criteria for considering studies for this review

#### Types of studies

Randomised clinical trials irrespective of publication year, status, and language were included.

#### Types of participants

Children and adolescents (<18 years old) with the diagnosis of type 1 diabetes (as defined by trialists) were included. In trials including both eligible participants (ie, <18 years) and ineligible participants (ie, >18 years), only separate data for children and adolescents were included.

#### Types of interventions

As experimental interventions we accepted regular human insulins, regardless of delivery method (syringe, pen or pump). As control interventions we accepted rapid-acting insulin analogues (insulin lispro, insulin aspart or insulin glulisine), regardless of method of delivery (syringe, pen or pump). We accepted any cointerventions, if these were planned to be delivered similarly in the experimental and control groups.

### Search strategy

To identify relevant trials, Cochrane Central Register of Controlled Trials (CENTRAL), Medical Literature Analysis and Retrieval System Online (MEDLINE), Excerpta Medica database (EMBASE), Latin American and Caribbean Health Sciences Literature (LI-LACS), Science Citation Index Expanded (SCI-EXPANDED), Conference Proceedings Citation Index—Science (CPCI-S) and the Health Technology Assessment (HTA) were searched. Additionally, searches were made in clinical trial registers, databases of clinical study reports and reference lists of relevant trial publications. The US Food and Drug Administration, the European Medicines Agency, relevant pharmaceutical companies and authors of included trials were contacted for unpublished trials or additional data. All databases were searched from their inception until 30 January 2026. For all detailed search strategies, see online supplemental text S1.

Three authors (JJP, PF and CDBS) independently screened relevant trials. Three authors (JJP, SJ and PF) independently (working in pairs) extracted data using a standardised data extraction sheet and assessed the risks of bias based on the Cochrane Risk of Bias tool, V.2 (RoB 2).^22 23^ Discrepancies were resolved through discussion, or, if required, through discussion with a third author (JCJ).

### Outcomes and subgroup analyses

Primary outcomes were severe hypoglycaemia, ketoacidosis and serious adverse events (as defined by the International Conference on Harmonisation-Good Clinical Practice (ICH-GCP)).^24^ Secondary outcomes were quality of life, haemoglobin A1c (HbA1c) and non-serious adverse events. Non-serious adverse events were classified as any adverse event not assessed as serious according to the ICH-GCP definition.^24^ Exploratory outcomes were all-cause mortality, postprandial blood glucose, continuous blood glucose monitoring, retinopathy, nephropathy, cardiovascular complications, hospitalisations, changes in height, changes in weight, quality of life of the parents/caregivers, cost of intervention and nocturnal hypoglycaemia. For all outcomes, the trial results reported at the longest follow-up were included. The following subgroup analyses were planned^13^:

- Trials at overall low risk of bias compared with trials at overall high risk of bias.
- Trials with for-profit bias compared with trials without for-profit bias.
- Trials with prepubertal participants (children) compared with trials with pubertal participants (adolescents).
- Type of comparator (insulin lispro, insulin aspart, insulin glulisine).
- Method of delivery (syringe, pen or pump).
- Trials performed in countries with high-income economies compared with trials performed in countries with middle- or low-income economies.

Subgroup analyses were considered exploratory, and they were conducted if more than two trials in different subgroups reported data on the outcome.

### Assessment of heterogeneity

Primarily, forest plots were investigated visually for signs of heterogeneity. Secondarily, statistical heterogeneity was assessed using I^2^ statistic^25^,^27^ and subgroup analyses (see above).

### Assessment of statistical and clinical significance

Meta-analyses were performed according to the Cochrane Handbook for Systematic Reviews of Interventions,^22^ Keus *et al*^28^ and the eight-step assessment by Jakobsen *et al.*^29^ Risk ratios (RR) were reported for dichotomous outcomes and mean difference (MD) for continuous outcomes. The threshold for statistical significance (p value of 0.025 or less as threshold)^29^ was adjusted per number of primary outcomes. Both random-effects (inverse variance and DerSimonian-Laird) and fixed-effect (Mantel-Haenszel) meta-analyses were performed for all analyses, and we primarily reported the most conservative results (highest p value). The least conservative results were considered sensitivity analyses.^22 29 30^ Trial sequential analysis was used to control for random errors.^31^,^39^ GRADE was used to assess the certainty of evidence for all outcomes.^40^,^42^ Fisher’s exact test was used to calculate p values for single trial results. Stata V.17 was used for all statistical analyses.^43^

If crossover trials reported data prior to crossover (ie, only including the first period of the trial with each participant providing data in either the intervention or the control group), the trial was treated as a parallel trial and these data were prioritised. If only data from the end of the trial after crossover were reported (ie, including both periods of the trial with each participant providing data in both the intervention and control group), data were included for short-term outcomes (severe hypoglycaemia, ketoacidosis, HbA1c, postprandial glucose level and nocturnal hypoglycaemia). For multi-arm parallel studies, we divided the number of events and the total number of participants evenly across the intervention groups to avoid double-counting in the meta-analyses.

## Results

### Study characteristics

Our literature search (last update: 30 January 2026) identified 15 127 records (online supplemental figure S1). Clinical study reports were obtained for three trials.^44^,^46^ 10 trials^4447^,^55^ randomising 1107 participants were included (online supplemental table S1). Six trials compared regular human insulins with insulin lispro,^47^,^52^ three trials compared regular human insulins with insulin aspart^53^,^55^ and one trial compared regular human insulins with insulin aspart and insulin lispro.^44^ All trials were performed in countries with high-income economies.^4447^,^56^ Eight trials included prepubertal participants,^47^,^4951^ one trial included pubertal participants^50^ and one trial included both.^44^

All trials were at high risk of bias (online supplemental table S2). The assessment time points ranged from 3 months^47^,^49^ to 6 months after the initiation of the intervention.^54^ None of the trials reported any data on retinopathy, nephropathy, cardiovascular complications, hospitalisations or costs of interventions.^4447^,^55^ No trials reported results suitable for meta-analyses on quality of life (child), height, weight or quality of life (parents/caregivers).^4447^,^55^ Trial definitions of outcomes are included in online supplemental table S3. Trial results not suitable for inclusion in meta-analyses are reported in online supplemental table S4.

The trial design was parallel in three trials^44 54 55^ and crossover in seven trials.^47^,^53^ Assessments of severe hypoglycaemia, ketoacidosis, HbA1c, postprandial glucose level and nocturnal hypoglycaemia were primarily based on data from crossover trials. In the crossover trials, participants received either regular human insulins or rapid-acting insulin analogues for the first 3–4 months.^47^,^53^ Afterwards, the participants received the other intervention for 3–4 months.^47^,^53^ Analyses only including parallel trial results and results of exploratory outcomes are provided in the supplementary material (online supplemental figures S18–S26, text S3).

No outcome included data from 10 or more trials. Therefore, no funnel plots were used to assess reporting bias, as specified in the protocol. No trials included both eligible participants and ineligible participants.

### Primary outcomes

#### Severe hypoglycaemia

Nine trials reported the outcome severe hypoglycaemia.^4447^,^54^ The assessment time points ranged from 3 months to 26 weeks after the initiation of the intervention.^4447^,^54^ A total of 30/762 (3.9%) experienced severe hypoglycaemia in the regular human insulin groups compared with 39/1007 (3.9%) in the rapid-acting insulin analogue groups.^4447^,^54^ Meta-analysis showed no evidence of a difference (RR 1.28, 95% CI 0.81 to 2.03; I^2^=0.0%; p=0.2851; nine trials; Bayes factor: 4.29) (online supplemental figure S2). Visual inspection of the forest plot and statistical tests (I^2^=0.0%) indicated no heterogeneity. Trial sequential analysis showed that the meta-analysis was underpowered (online supplemental figure S3). This outcome result was assessed at high risk of bias, and the certainty of the evidence was very low (online supplemental tables S2 and S5).

Tests of interaction comparing the effects of age (p=0.909), insulin lispro versus insulin aspart (p=0.744) and method of delivery (p=0.788) showed no evidence of differences (online supplemental figures S4–S6). The remaining predefined subgroup analyses could not be performed due to a lack of relevant data.

#### Ketoacidosis

Two trials reported the outcome ketoacidosis.^44 52^ The assessment time points ranged from 16 weeks to 26 weeks after the initiation of the intervention.^44 52^ A total of 4/123 (3.3%) experienced ketoacidosis in the regular human insulin groups compared with 12/309 (3.9%) in the rapid-acting insulin analogue groups.^44 52^ Meta-analysis showed no evidence of a difference (RR 0.88, 95% CI 0.26 to 2.93; I^2^=0.0%; p=0.8593; two trials; Bayes factor: 0.99) (online supplemental figure S7). Visual inspection of the forest plot and statistical tests (I^2^=0.0%) indicated no heterogeneity. Trial sequential analysis showed that the meta-analysis was underpowered (no graph produced). This outcome result was assessed at high risk of bias, and the certainty of the evidence was very low (online supplemental tables S2 and S5).

Tests of interaction comparing the effects of age (p=0.183), insulin lispro versus insulin aspart (p=0.342) and method of delivery (p=0.183) showed no evidence of differences (online supplemental figures S8–S10). The remaining predefined subgroup analyses could not be performed due to a lack of relevant data.

#### Serious adverse events

Two trials reported the outcome serious adverse events.^44 54^ The individual serious adverse events were not described.^44 54^ Four additional trials assessed the outcome adverse events, but their data could not be included in the analyses (online supplemental table S4).^47 48 50 53^ The assessment time points ranged from 24 weeks to 26 weeks after the initiation of the intervention.^44 54^ A total of 7/117 (6.0%) experienced a serious adverse event in the regular human insulin groups compared with 21/302 (7.0%) in the rapid-acting insulin analogue groups.^44 54^ Meta-analysis showed no evidence of a difference (RR 1.00, 95% CI 0.44 to 2.25; I^2^=0.0%; p=0.9958; two trials; Bayes factor: 1.15) (online supplemental figure S11). Visual inspection of the forest plot and statistical tests (I^2^=0.0%) indicated no heterogeneity. Trial sequential analysis showed that the meta-analysis was underpowered (no graph produced). This outcome result was assessed at high risk of bias, and the certainty of the evidence was very low (online supplemental tables S2 and S5).

Tests of interaction comparing the effects of age (p=0.258) and insulin lispro versus insulin aspart (p=0.700) showed no evidence of differences (online supplemental figures S12 and S13). The remaining predefined subgroup analyses could not be performed due to a lack of relevant data.

### Secondary outcomes

#### Haemoglobin A1c

Seven trials reported the outcome HbA1c.^4447^,^51 54^ Three additional trials assessed the outcome HbA1c, but their data could not be included in the analyses (online supplemental table S4).^52 53 55^ The assessment time points ranged from 3 months to 26 weeks after the initiation of the intervention.^4447^,^51 54^ Meta-analysis showed no evidence of a difference (MD −0.05, 95% CI −0.17 to 0.07; I^2^=21.3%; p=0.3957; seven trials) (online supplemental figure S14). Visual inspection of the forest plot and statistical tests (I^2^=21.3%) indicated little heterogeneity. Trial sequential analysis confirmed the meta-analysis result (online supplemental figure S15). This outcome result was assessed at high risk of bias, and the certainty of the evidence was very low (online supplemental tables S2 and S5).

Tests of interaction comparing the effects of age (p=0.115) and insulin lispro versus insulin aspart (p=0.680) showed no evidence of differences (online supplemental figures S4–S6). The remaining predefined subgroup analyses could not be performed due to a lack of relevant data.

#### Non-serious adverse events

One trial reported the outcome non-serious adverse events.^54^ Five additional crossover trials assessed the outcome adverse events without specifying whether they were serious or non-serious, and the data were not reported in a way suitable for meta-analysis (eg, it was unclear in which group the events occurred). Therefore, these data have been summarised descriptively in online supplemental table S4.^44 47 48 50 53^ The assessment time point was 26 weeks after the initiation of the intervention.^54^ A total of 15/21 (71.4%) experienced a non-serious adverse event in the regular human insulin group compared with 10/20 (50.0%) in the rapid-acting insulin analogue group (Fisher’s exact test: p=0.2082).^54^ This outcome result was assessed at high risk of bias, and the certainty of the evidence was very low (online supplemental table S2 and S5).

### Patient and public involvement

Patients and the public were not involved in the design, conduct or reporting of our research. The patient and public will be involved in the dissemination of our research.

## Discussion

### Principal findings

We conducted a systematic review assessing the beneficial and harmful effects of regular human insulins versus rapid-acting insulin analogues in children and adolescents with type 1 diabetes. 10 trials randomising 1107 participants were included. All results were high risk of bias, and the certainty of the evidence was very low. Meta-analyses showed no evidence of a difference between regular human insulins and rapid-acting insulin analogues on severe hypoglycaemia, ketoacidosis and serious adverse events. Trial sequential analysis showed that all meta-analyses of primary outcomes were underpowered. The required information size was far from reached, indicating that clinically important differences cannot be excluded.

### Strengths and weaknesses of the study

This systematic review has several strengths. The methodology was thoroughly described in a published protocol before initiating the literature searches,^13^ and it was based on the eight-step assessment suggested by Jakobsen *et al*^57^ and trial sequential analysis.^31^ The methodological quality of the included trials was assessed with up-to-date methods.^57^,^59^ Risk of bias was assessed using the RoB 2,^58^ and the overall certainty of the evidence was assessed using GRADE.^59^ Therefore, both risks of random errors (‘play of chance’) and systematic errors (bias) were considered in the present review.

This systematic review also has limitations. First, most of the included trials were crossover trials.^47^,^53^ Seven trials did not report separate data for the first period of the trial.^47^,^53^ Therefore, data from both periods of these trials were included in the analyses for short-term outcomes (severe hypoglycaemia, ketoacidosis, HbA1c, postprandial glucose level and nocturnal hypoglycaemia). This creates a risk of carry-over effect, where the treatment from the first period could affect the measurements in the second period of the trial.^60^ This risk is lower for short-term outcomes like hypoglycaemia but higher for HbA1c.^60^ Also, this makes it impossible to assess the long-term effect of the interventions in crossover trials, as it remains unclear whether the first or the second intervention caused the outcome.^60^ Diabetes is associated with multiple long-term complications related to microvascular diseases (retinopathy, nephropathy and neuropathy) and macrovascular diseases (cardiovascular, cerebrovascular and peripheral vascular diseases). When these relevant long-term outcomes cannot be assessed in crossover trials, it is difficult to accurately compare the clinically relevant effects of the interventions based on the included trials. For short-term outcomes, data from crossover trials were included by considering these as if they were two separate parallel-group studies. However, this approach does not account for within-person correlation and may introduce bias.^61^

Second, all trials had methodological limitations and were assessed at high risk of bias. This was primarily driven by lack of blinding, missing data, poor reporting and no predefined methodology in protocols, lack of clinical trial registrations or statistical analysis plans.^4447^,^55^ Therefore, the results may be inaccurate.^62^,^64^ Also, the certainty of the evidence was very low for all outcomes. In addition to the risk of bias, this was primarily driven by downgrading due to indirectness and imprecision. All trials were performed in high-income economies, meaning the population of the trials do not necessarily represent the population which are expected to receive the interventions.^2^ When the results from these trials are extrapolated to other populations, the observed beneficial and harmful effects may be different than expected.^65^ In addition, all included trials were conducted before the widespread adoption of continuous glucose monitoring and contemporary insulin delivery strategies, which may limit applicability to modern clinical practice. Also, due to the small amount of available data, all meta-analyses were underpowered, and due to the limited number of studies, results of subgroup analyses should be interpreted with caution. In future trials, these methodological considerations should be incorporated in the trial design by predefining the methodology in publicly available protocols, ensuring blinding and including broader populations to increase the internal and external validity of the trials.

Third, all trials were funded by the industry.^4447^,^55^ Hence, there is a risk that our results overestimate the beneficial effects due to for-profit bias.^66^ Since no trials were without affiliations to the industry, it was not possible to investigate such an effect through subgroup analyses.

Fourth, another limitation of this review is the lack of identification of grey literature. Although several attempts were made to obtain unpublished data, only three clinical study reports were included. It is possible that gaining access to additional data could change the results of the review.

Fifth, none of the trials reported any data on retinopathy, nephropathy, cardiovascular complications, hospitalisations or cost of interventions.^4447^,^55^ No trials reported results suitable for meta-analyses on quality of life (child), height, weight or quality of life (parents/caregivers).^4447^,^55^

Lastly, the included trials differed in population, intervention, timing of intervention and definition and measurement of outcomes. This clinical heterogeneity could potentially limit the interpretability of pooled results. However, several subgroup and sensitivity analyses were conducted, and no clear evidence of statistical heterogeneity was identified

### Strengths, weaknesses and results in relation to other studies

Two reviews have previously assessed regular human insulins and rapid-acting insulin analogues for children and adolescents with type 1 diabetes.^18 19^ Neither review found differences in glycaemic control or hypoglycaemic episodes between regular human insulins and rapid-acting insulin analogues.^18 19^ However, both reviews included fewer studies and did not employ trial sequential analysis methods to control for random errors, nor did they formally assess risk of bias or the certainty of evidence using GRADE. Hence, the current review provides a more comprehensive and methodologically rigorous synthesis of the available evidence.

A review from 2020 assessed regular human insulins versus rapid-acting insulin analogues in adults with type 1 diabetes.^15^ This review showed beneficial effects of rapid-acting insulin analogues compared with regular human insulins on postprandial glucose and HbA1c but did not report any results on severe hypoglycaemia, ketoacidosis or serious adverse events.^15^ Differences in meal patterns, insulin sensitivity during growth and caregiver-driven dosing may attenuate pharmacokinetic advantages observed in adults. Unlike the previous review from 2020, this current study did not find evidence of a difference between regular human insulins and rapid-acting insulin analogues in children and adolescents. This may reflect the limited available data and resulting imprecision, or it may indicate that the two interventions have comparable effects.

### Implications for clinicians, policymakers and future research

This systematic review found no evidence of a difference between regular human insulin and rapid-acting insulin analogues on clinical outcomes. In practice, given the likely similar effects on outcomes such as HbA1c and hypoglycaemia, other factors, including cost, availability and patient or caregiver preferences, may guide future clinical decision-making. These findings are particularly relevant in settings where regular human insulin remains substantially less expensive and more widely available

## Conclusion

Current research shows no differential effects between regular human insulin and rapid-acting insulin analogues for children and adolescents with type 1 diabetes, but the evidence is very uncertain. More randomised clinical trials are needed to assess the comparative effects.

### Differences between the protocol and the review

We decided to include data from both periods from cross-over trials for short-term outcomes (severe hypoglycaemia, ketoacidosis, HbA1c, postprandial glucose level and nocturnal hypoglycaemia).

## Supplementary material

10.1136/bmjopen-2025-100186 online supplemental file 1

## Data Availability

Data are available in a public, open access repository.
