# Supplementary material for: Regular human insulins versus rapid-acting insulin analogues in children and adolescents with type 1 diabetes: a systematic review with meta-analysis and trial sequential analysis
Source: BMJ Open. 2026 May 14;16(5):e100186. doi: 10.1136/bmjopen-2025-100186 (PMC13182422; doi:10.1136/bmjopen-2025-100186)
Supplement: online supplemental file 1 [file bmjopen-16-5-s001.pdf]

Supplementary material for regular human insulins versus rapid-acting insulin analogues in children and adolescents with type 1 diabetes: a systematic review with meta-analysis and Trial Sequential Analysis

Johanne Juul Petersen, BSc *[1 2], Pascal Faltermeier, MSc [1], Sophie Juul, PhD [1 3 4], Caroline Barkholt Kamp, MSc [1 5], Christina Dam Bjerregaard Sillassen, MD [1 5 6], Tiago Jeronimo Dos Santos PhD [7 8], Janus Christian Jakobsen, Dr.Med.Sci [1 5]

Affiliations

[1] Copenhagen Trial Unit, Centre for Clinical Intervention Research, Rigshospitalet, Capital Region of Denmark, Copenhagen, Denmark

[2] Faculty of Health and Medical Sciences, University of Copenhagen, Copenhagen, Denmark

[3] Stolpegaard Psychotherapy Centre, Mental Health Services in the Capital Region of Denmark, Gentofte, Denmark

[4] Department of Psychology, University of Copenhagen, Copenhagen, Denmark

[5] Department of Regional Health Research, The Faculty of Health Sciences, University of Southern Denmark, Odense, Denmark

[6] Department of Cardiology and Endocrinology, Slagelse Hospital, Region of Zealand, Slagelse, Denmark

[7] Unit of Pediatrics, Hospital Vithas Almería, Instituto Hispalense de Pediatría, Almería, Andalusia, Spain

[8] Department of Nursing, Physiotherapy, and Medicine, Faculty of Health Sciences, University of Almería, Almería, Spain

Contact information:

* Corresponding author: Johanne Juul Petersen: johanne.juul.petersen@ctu.dk, +4528933035, Copenhagen Trial Unit, Centre for Clinical Intervention Research, Rigshospitalet, Capital Region of Denmark

Indholdsfortegnelse

Supplementary figures 5

Figure S1: PRISMA flowchart 5
Figure S2: Meta-analysis of regular human insulins (RHI) versus rapid-acting insulin analogues (RAIA) on severe hypoglycemia 6
Figure S3: Trial Sequential Analysis of regular human insulins (RHI) versus rapid-acting insulin analogues (RAIA) on severe hypoglycemia 7
Figure S4: Subgroup analysis on age of regular human insulins (RHI) versus rapid-acting insulin analogues (RAIA) on severe hypoglycemia 8
Figure S5: Subgroup analysis on insulin lispro versus insulin aspart of regular human insulins (RHI) versus rapid-acting insulin analogues (RAIA) on severe hypoglycemia 9
Figure S6: Subgroup analysis on method of delivery of regular human insulins (RHI) versus rapid-acting insulin analogues (RAIA) on severe hypoglycemia 10
Figure S7: Meta-analysis of regular human insulins (RHI) versus rapid-acting insulin analogues (RAIA) on ketoacidosis 11
Figure S8: Subgroup analysis on age of regular human insulins (RHI) versus rapid-acting insulin analogues (RAIA) on ketoacidosis 12
Figure S9: Subgroup analysis on insulin lispro versus insulin aspart of regular human insulins (RHI) versus rapid-acting insulin analogues (RAIA) on ketoacidosis 13
Figure S10: Subgroup analysis on method of delivery of regular human insulins (RHI) versus rapid-acting insulin analogues (RAIA) on ketoacidosis 14
Figure S11: Meta-analysis of regular human insulins (RHI) versus rapid-acting insulin analogues (RAIA) on serious adverse events 15
Figure S12: Subgroup analysis on age of regular human insulins (RHI) versus rapid-acting insulin analogues (RAIA) on serious adverse events 16
Figure S13: Subgroup analysis on insulin lispro versus insulin aspart of regular human insulins (RHI) versus rapid-acting insulin analogues (RAIA) on serious adverse events 17
Figure S14: Meta-analysis of regular human insulins (RHI) versus rapid-acting insulin analogues (RAIA) on HbA1c 18
Figure S15: Trial Sequential Analysis of regular human insulins (RHI) versus rapid-acting insulin analogues (RAIA) on HbA1c 19
Figure S16: Subgroup analysis on age of regular human insulins (RHI) versus rapid-acting insulin analogues (RAIA) on HbA1c 20
Figure S17: Subgroup analysis on insulin lispro versus insulin aspart of regular human insulins (RHI) versus rapid-acting insulin analogues (RAIA) on HbA1c 21
Figure S18: Fixed-effects meta-analysis of regular human insulins (RHI) versus rapid-acting insulin analogues (RAIA) on postprandial glucose level 22
Figure S19: Random-effects meta-analysis of regular human insulins (RHI) versus rapid-acting insulin analogues (RAIA) on postprandial glucose level 23
Figure S20: Trial Sequential Analysis of regular human insulins (RHI) versus rapid-acting insulin analogues (RAIA) on postprandial glucose level 24
Figure S21: Subgroup analysis on age of regular human insulins (RHI) versus rapid-acting insulin analogues (RAIA) on postprandial glucose level 25
Figure S22: Subgroup analysis on insulin lispro versus insulin aspart of regular human insulins (RHI) versus rapid-acting insulin analogues (RAIA) on postprandial glucose level 26
Figure S23: Subgroup analysis on method of delivery of regular human insulins (RHI) versus rapid-acting insulin analogues (RAIA) on postprandial glucose level 27
Figure S24: Meta-analysis of regular human insulins (RHI) versus rapid-acting insulin analogues (RAIA) on severe hypoglycemia (parallel trials) 28
Figure S25: Meta-analysis of regular human insulins (RHI) versus rapid-acting insulin analogues (RAIA) on HbA1c (parallel trials) 29
Figure S26: Trial Sequential Analysis of regular human insulins (RHI) versus rapid-acting insulin analogues (RAIA) on HbA1c (parallel trials) 30

Supplementary tables **31**
Table S1: Characteristics of included trials 31
Table S2: Risk of bias 35
Table S3: Trial definitions of outcomes 36
Table S4: Trial results not suitable for meta-analyses 39
Table S5: Summary of findings table 43

Supplementary text **44**
Text S1: PRISMA checklist 44
Text S2: Search strategy 49
Text S3: Supplementary results 53

Supplementary figures

Figure S1: PRISMA flowchart

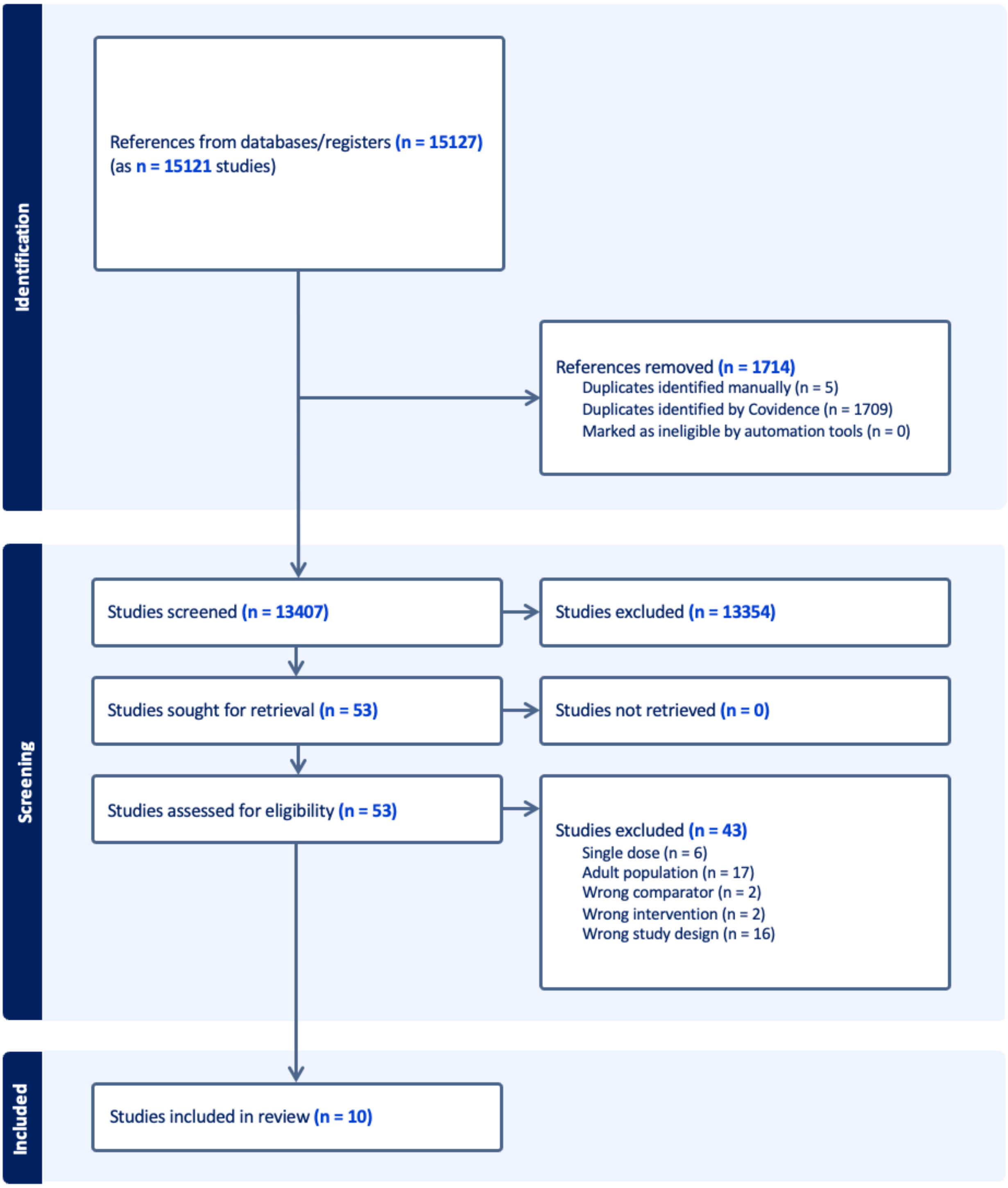

Figure S2: Meta-analysis of regular human insulins (RHI) versus rapid-acting insulin analogues (RAIA) on severe hypoglycemia

Study	RHI Event	RHI No event	RAIA Event	RAIA No event	Risk ratio with 95% CI	Weight (%)
Fairchild 2000	2	33	1	34	2.00 [0.19, 21.06]	3.49
Deeb 2001a	3	27	2	57	2.95 [0.52, 16.71]	4.70
Deeb 2001b	3	26	3	56	2.03 [0.44, 9.46]	6.89
Tupola 2001	2	20	2	20	1.00 [0.15, 6.48]	6.97
Holcombe 2002	5	452	5	452	1.00 [0.29, 3.43]	17.43
Ford-Adams 2003	1	21	2	20	0.50 [0.05, 5.12]	6.97
Tubiana-Rufi 2004	2	25	2	25	1.00 [0.15, 6.59]	6.97
ANA-2126 (Aspart)	5	43	11	176	1.77 [0.65, 4.85]	15.67
ANA-2126 (Lispro)	4	44	8	87	0.99 [0.31, 3.12]	18.72
Danne 2007	3	20	2	22	1.57 [0.29, 8.53]	6.82
Pankowska 2010	0	21	1	19	0.32 [0.01, 7.38]	5.35
**Overall**					1.28 [0.81, 2.03]	

Heterogeneity: $I^2 = 0.00\%$, $H^2 = 1.00$

Test of $\theta_i = \theta_j$: $Q(10) = 3.69$, $p = 0.96$

Test of $\theta = 0$: $z = 1.07$, $p = 0.29$

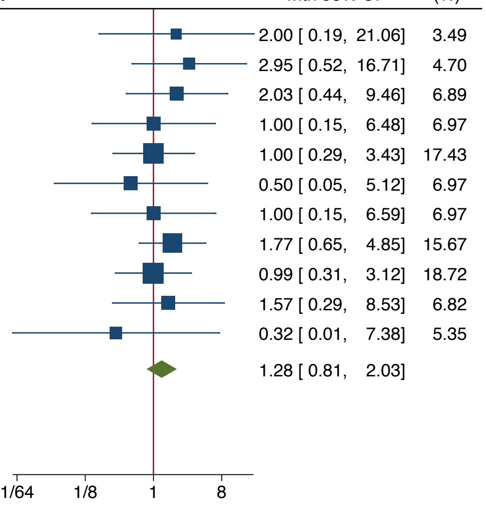

Fixed-effects Mantel–Haenszel model

Figure S3: Trial Sequential Analysis of regular human insulins (RHI) versus rapid-acting insulin analogues (RAIA) on severe hypoglycemia

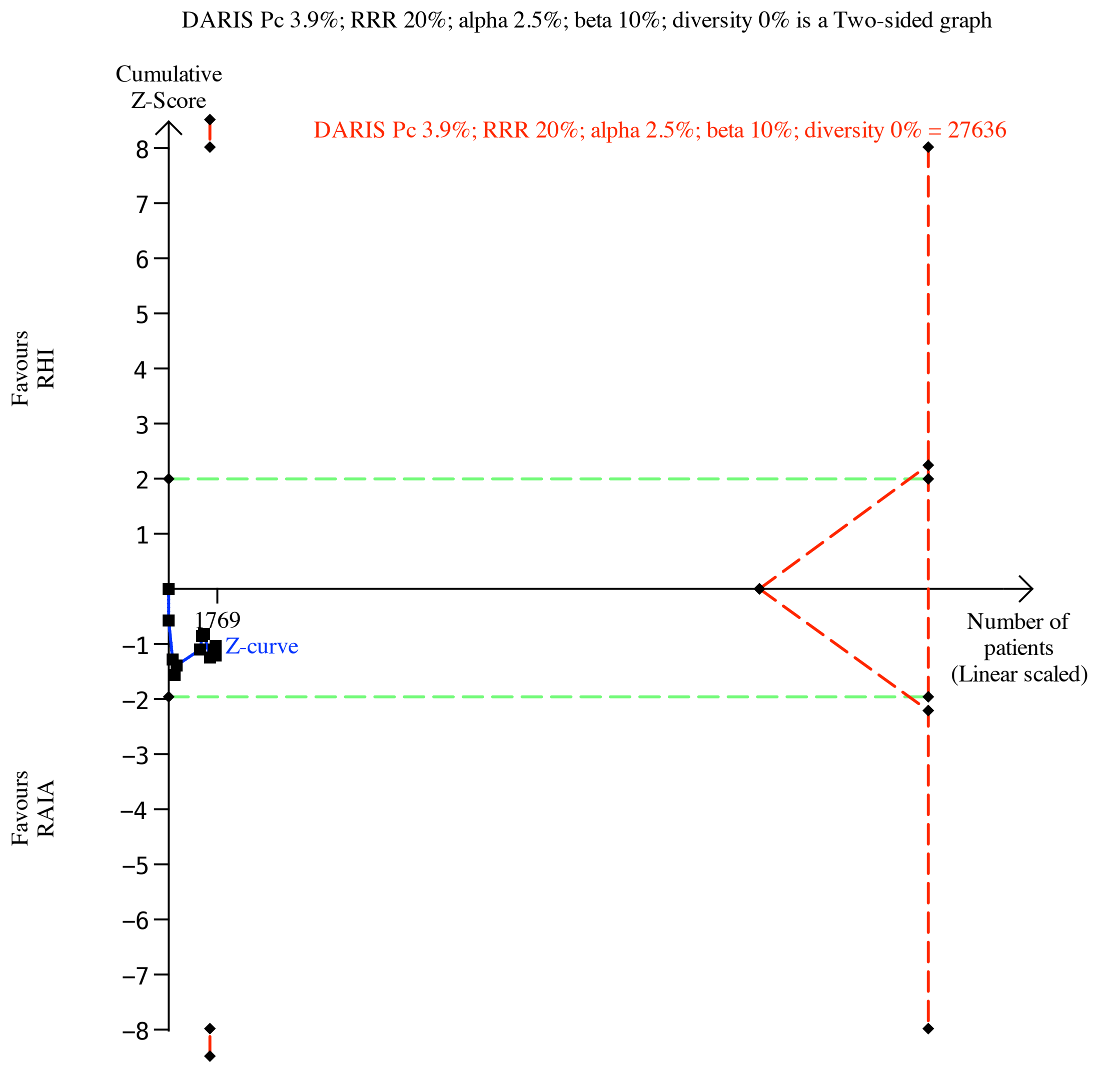

Figure S4: Subgroup analysis on age of regular human insulins (RHI) versus rapid-acting insulin analogues (RAIA) on severe hypoglycemia

Study	RHI Event	RHI No event	RAIA Event	RAIA No event
**Prepubertal**				
Fairchild 2000	2	33	1	34
Deeb 2001a	3	27	2	57
Deeb 2001b	3	26	3	56
Tupola 2001	2	20	2	20
Ford-Adams 2003	1	21	2	20
Tubiana-Rufi 2004	2	25	2	25
Danne 2007	3	20	2	22
Pankowska 2010	0	21	1	19
Heterogeneity: $I^2 = 0.00\%$, $H^2 = 1.00$				
Test of $\theta_i = \theta_j$: $Q(7) = 2.90$, $p = 0.89$				
Test of $\theta = 0$: $z = 0.88$, $p = 0.38$				
**Prepubertal and pubertal**				
ANA-2126 (Aspart)	5	43	11	176
ANA-2126 (Lispro)	4	44	8	87
Heterogeneity: $I^2 = 0.00\%$, $H^2 = 1.00$				
Test of $\theta_i = \theta_j$: $Q(1) = 0.56$, $p = 0.45$				
Test of $\theta = 0$: $z = 0.77$, $p = 0.44$				
**Pubertal**				
Holcombe 2002	5	452	5	452
Heterogeneity: $I^2 = 0.00\%$, $H^2 = 1.00$				
Test of $\theta_i = \theta_j$: $Q(0) = 0.00$, $p = .$				
Test of $\theta = 0$: $z = 0.00$, $p = 1.00$				
**Overall**				
Heterogeneity: $I^2 = 0.00\%$, $H^2 = 1.00$				
Test of $\theta_i = \theta_j$: $Q(10) = 3.69$, $p = 0.96$				
Test of $\theta = 0$: $z = 1.07$, $p = 0.29$				
Test of group differences: $Q_b(2) = 0.19$, $p = 0.91$				

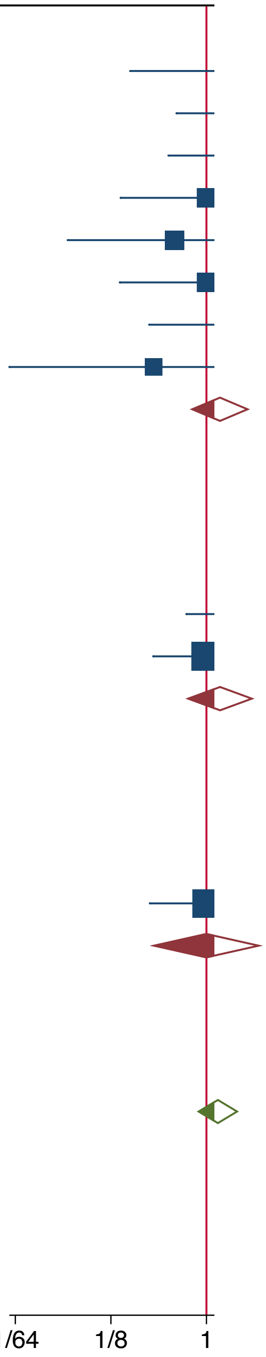

Fixed-effects Mantel–Haenszel model

Figure S5: Subgroup analysis on insulin lispro versus insulin aspart of regular human insulins (RHI) versus rapid-acting insulin analogues (RAIA) on severe hypoglycemia

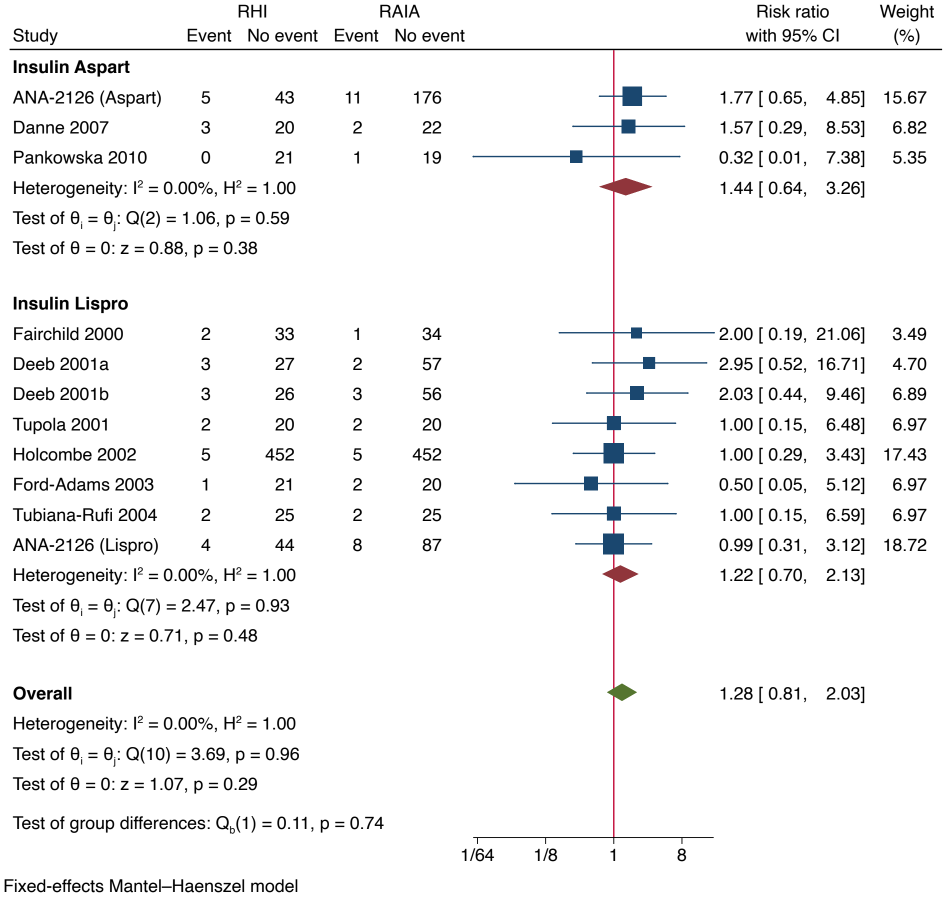

Figure S6: Subgroup analysis on method of delivery of regular human insulins (RHI) versus rapid-acting insulin analogues (RAIA) on severe hypoglycemia

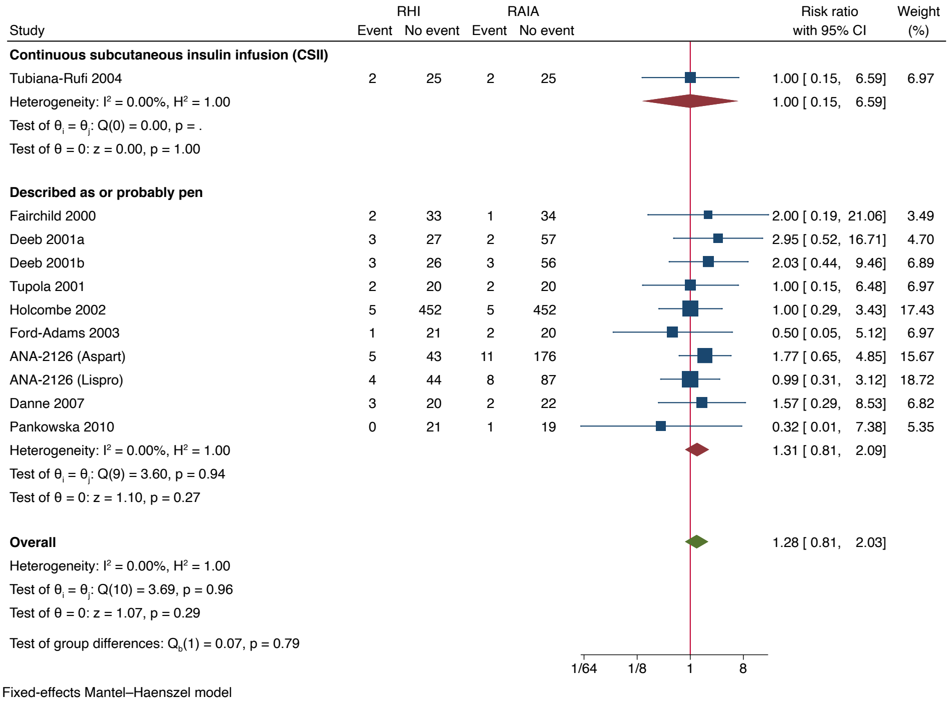

Figure S7: Meta-analysis of regular human insulins (RHI) versus rapid-acting insulin analogues (RAIA) on ketoacidosis

Study	RHI Event	RHI No event	RAIA Event	RAIA No event	Risk ratio with 95% CI	Weight (%)
Tubiana-Rufi 2004	2	25	0	27	5.00 [0.25, 99.51]	8.08
ANA-2126 (Aspart)	1	47	9	178	0.43 [0.06, 3.33]	59.39
ANA-2126 (Lispro)	1	47	3	92	0.66 [0.07, 6.17]	32.53
**Overall**					0.88 [0.26, 2.93]	

Heterogeneity: I^2 = 0.00%, H^2 = 1.00

Test of $\theta_i = \theta_j$: Q(2) = 1.82, p = 0.40

Test of θ = 0: z = -0.22, p = 0.83

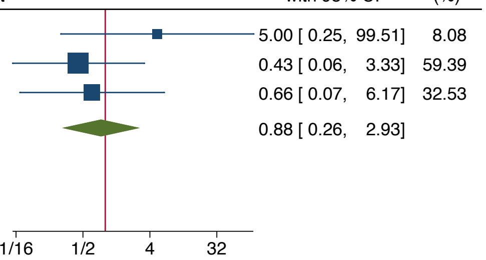

Fixed-effects Mantel–Haenszel model

Figure S8: Subgroup analysis on age of regular human insulins (RHI) versus rapid-acting insulin analogues (RAIA) on ketoacidosis

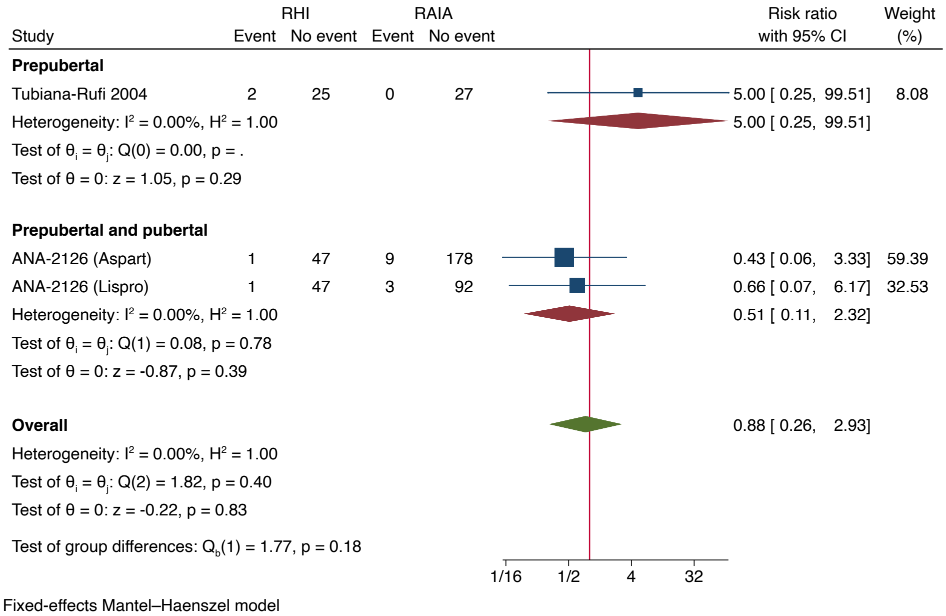

Figure S9: Subgroup analysis on insulin lispro versus insulin aspart of regular human insulins (RHI) versus rapid-acting insulin analogues (RAIA) on ketoacidosis

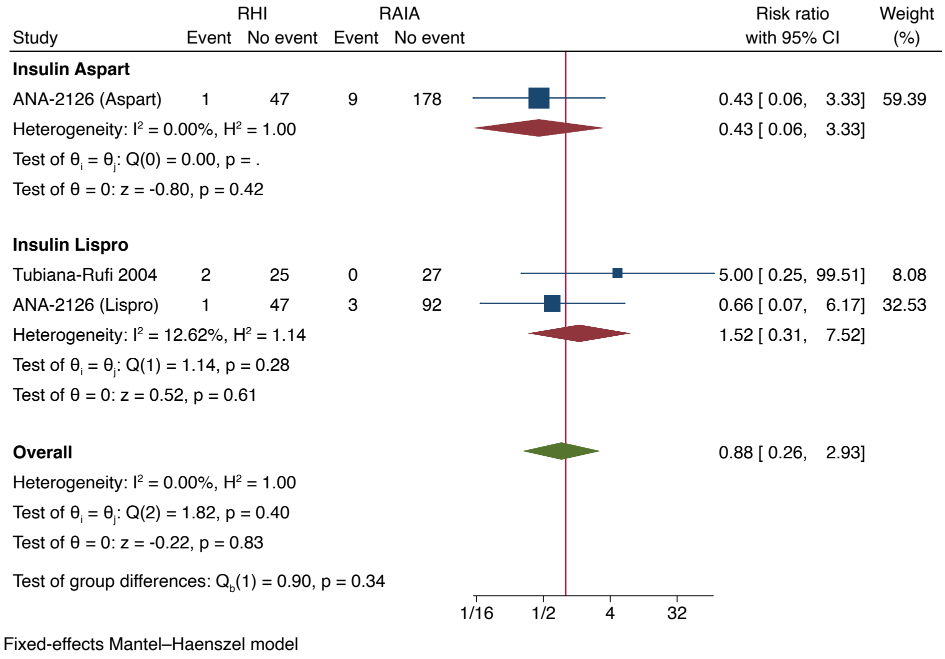

Figure S10: Subgroup analysis on method of delivery of regular human insulins (RHI) versus rapid-acting insulin analogues (RAIA) on ketoacidosis

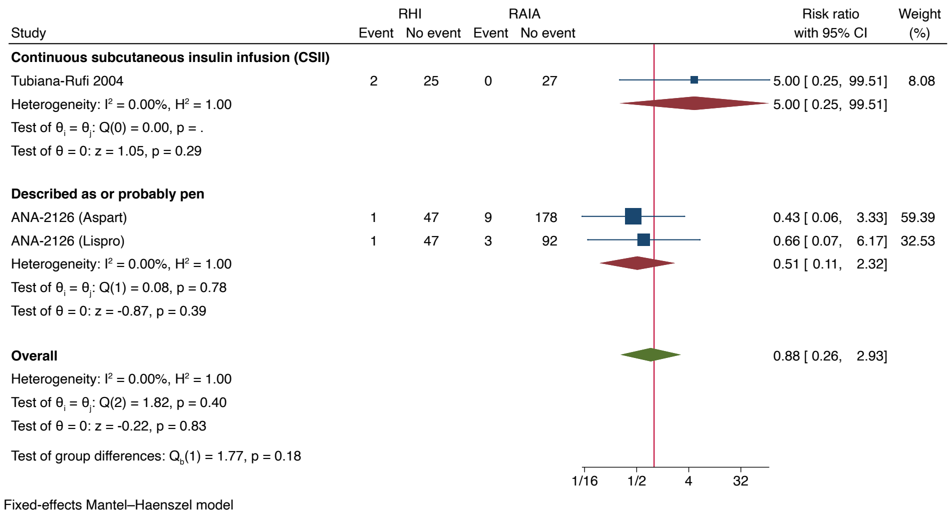

Figure S11: Meta-analysis of regular human insulins (RHI) versus rapid-acting insulin analogues (RAIA) on serious adverse events

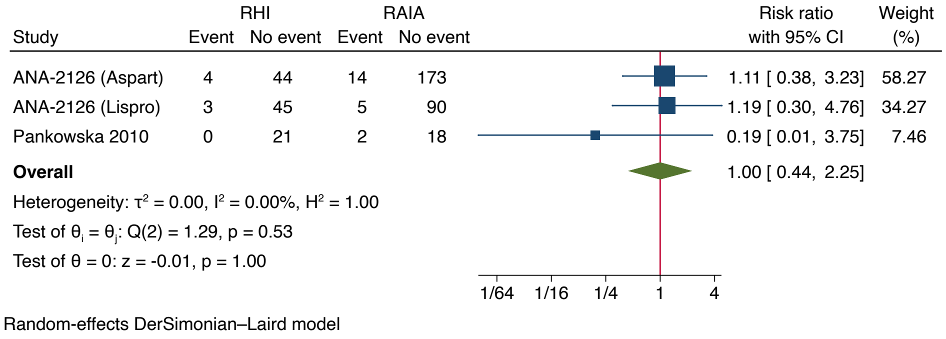

Figure S12: Subgroup analysis on age of regular human insulins (RHI) versus rapid-acting insulin analogues (RAIA) on serious adverse events

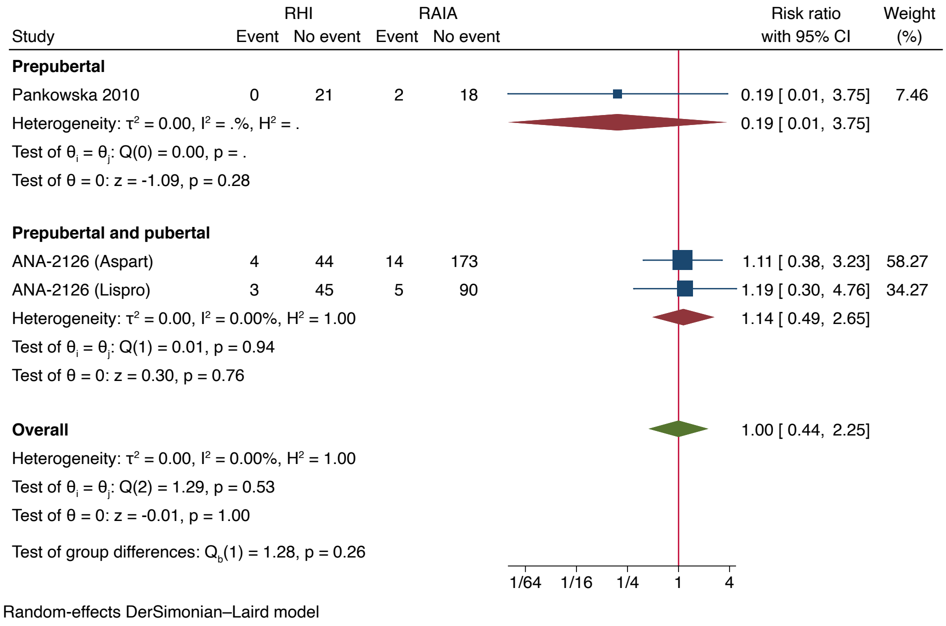

Figure S13: Subgroup analysis on insulin lispro versus insulin aspart of regular human insulins (RHI) versus rapid-acting insulin analogues (RAIA) on serious adverse events

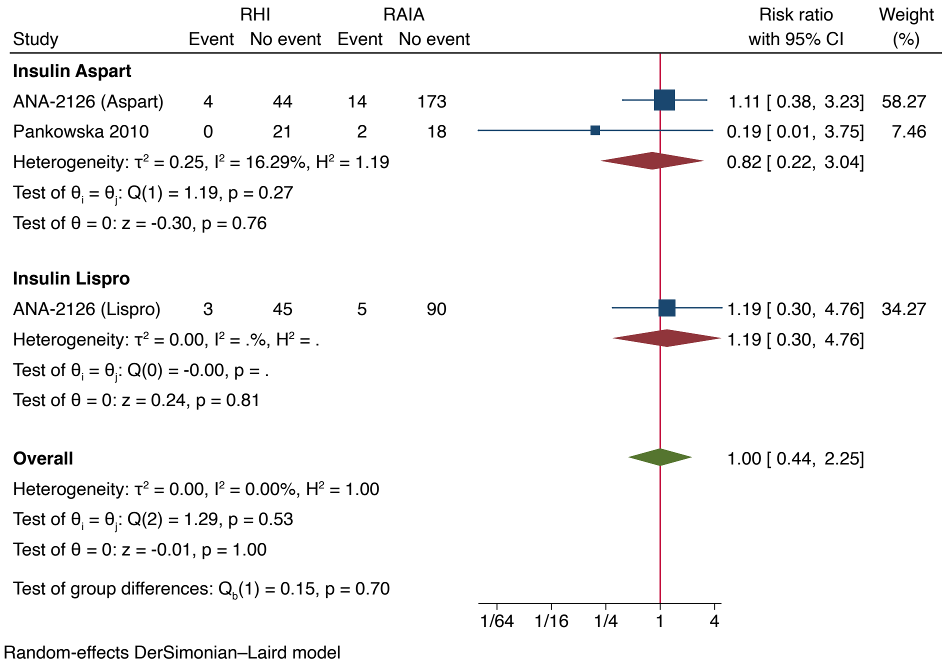

Figure S14: Meta-analysis of regular human insulins (RHI) versus rapid-acting insulin analogues (RAIA) on HbA1c

Study	RHI N	RHI Mean	RHI SD	Rapid-acting insulin analogues N	Rapid-acting insulin analogues Mean	Rapid-acting insulin analogues SD	Mean diff. with 95% CI	Weight (%)
Fairchild 2000	35	8.14	.77	35	8.33	.89	-0.19 [-0.58, 0.20]	9.74
Deeb 2001a	30	8.43	1	59	8.4	1.1	0.03 [-0.44, 0.50]	6.72
Deeb 2001b	29	8.43	1	59	8.54	1	-0.11 [-0.55, 0.33]	7.49
Tupola 2001	22	-.4	.7	22	.2	.8	-0.60 [-1.04, -0.16]	7.50
Holcombe 2002	457	8.7	1.65	457	8.69	1.52	0.01 [-0.20, 0.22]	34.99
Ford-Adams 2003	12	8.4	1	11	8.9	1	-0.50 [-1.32, 0.32]	2.21
ANA-2126 (Aspart)	48	.1	1	187	.1	1	0.00 [-0.32, 0.32]	14.72
ANA-2126 (Lispro)	48	.1	1	95	-.1	1	0.20 [-0.15, 0.55]	12.29
Pankowska 2010	21	7.6	1	20	7.6	.9	0.00 [-0.58, 0.58]	4.35
**Overall**							-0.05 [-0.17, 0.07]	

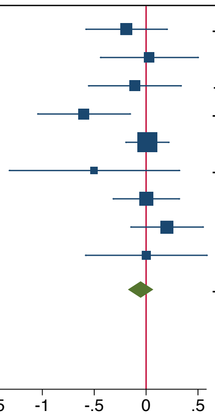

Heterogeneity: I^2 = 21.34%, H^2 = 1.27

Test of $\theta_i = \theta_j$: Q(8) = 10.17, p = 0.25

Test of θ = 0: z = -0.85, p = 0.40

Fixed-effects inverse-variance model

Figure S15: Trial Sequential Analysis of regular human insulins (RHI) versus rapid-acting insulin analogues (RAIA) on HbA1c

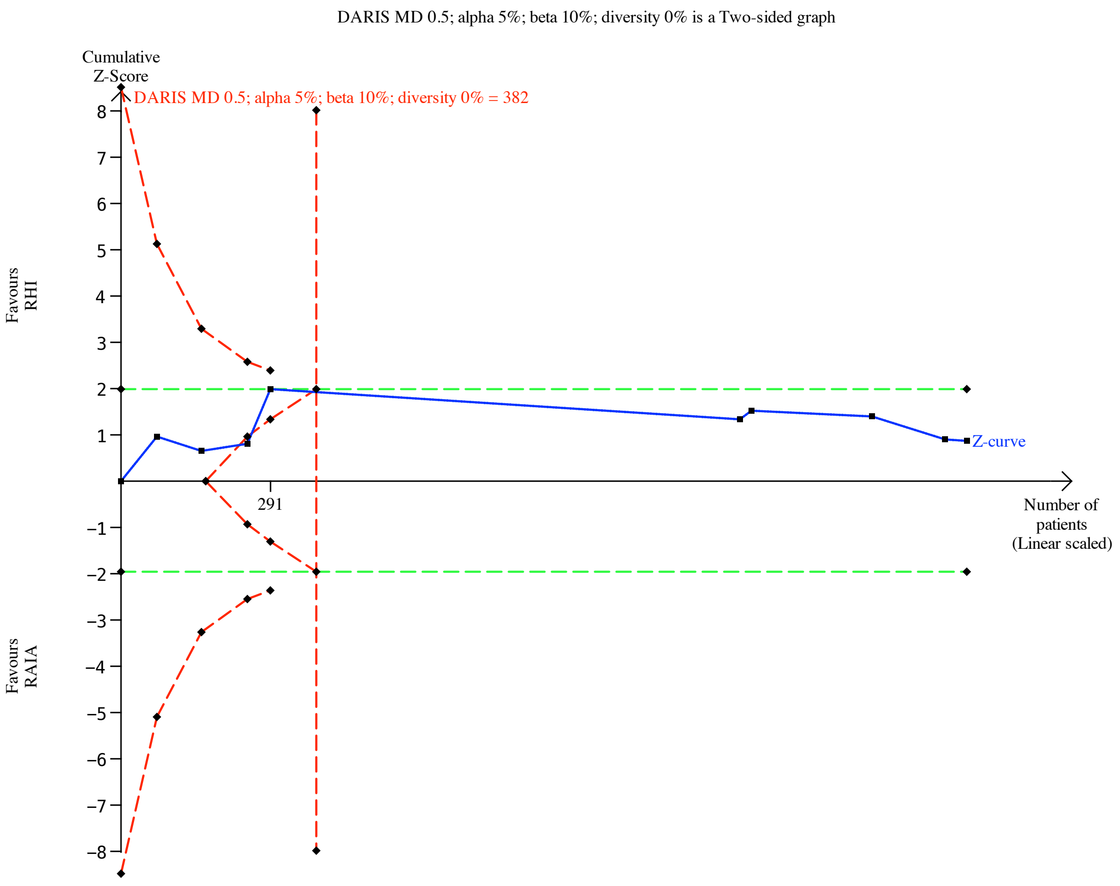

Figure S16: Subgroup analysis on age of regular human insulins (RHI) versus rapid-acting insulin analogues (RAIA) on HbA1c

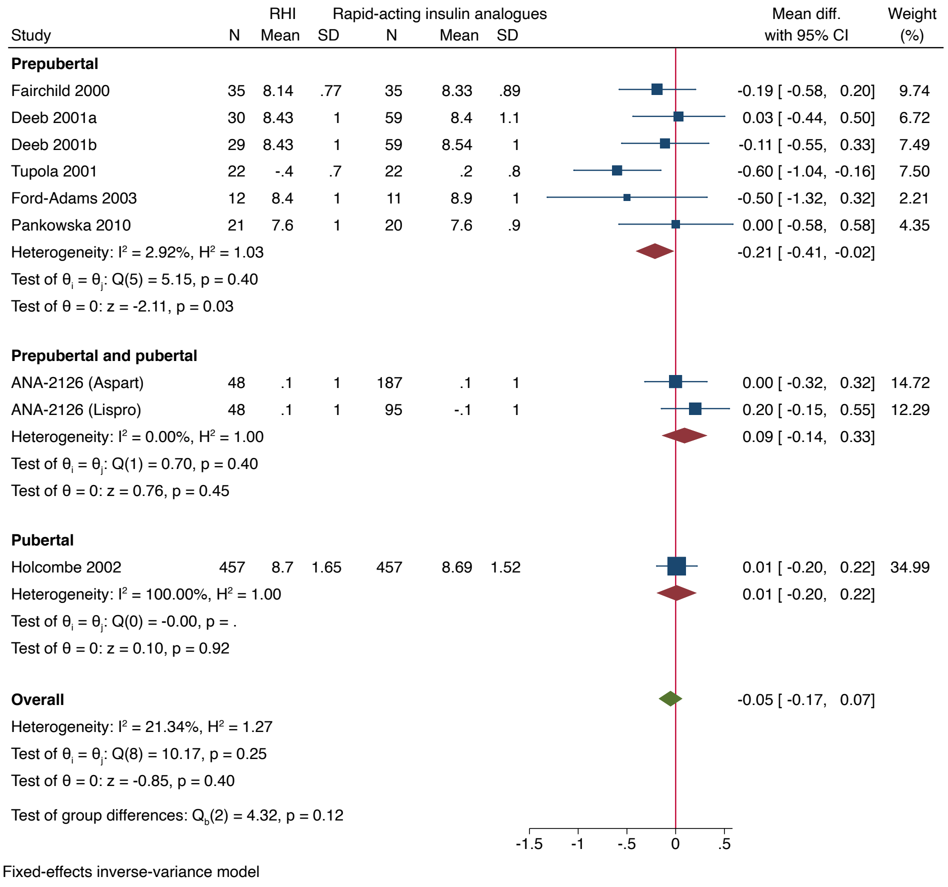

Figure S17: Subgroup analysis on insulin lispro versus insulin aspart of regular human insulins (RHI) versus rapid-acting insulin analogues (RAIA) on HbA1c

Study	RHI N	RHI Mean	RHI SD	Rapid-acting insulin analogues N	Rapid-acting insulin analogues Mean	Rapid-acting insulin analogues SD	Mean diff. with 95% CI	Weight (%)
**Insulin Aspart**								
ANA-2126 (Aspart)	48	.1	1	187	.1	1	0.00 [-0.32, 0.32]	14.72
Pankowska 2010	21	7.6	1	20	7.6	.9	0.00 [-0.58, 0.58]	4.35
Heterogeneity: $I^2 = 0.00\%$, $H^2 = 1.00$							0.00 [-0.28, 0.28]	
Test of $\theta_i = \theta_j$: Q(1) = 0.00, p = 1.00								
Test of θ = 0: z = 0.00, p = 1.00								
**Insulin Lispro**								
Fairchild 2000	35	8.14	.77	35	8.33	.89	-0.19 [-0.58, 0.20]	9.74
Deeb 2001a	30	8.43	1	59	8.4	1.1	0.03 [-0.44, 0.50]	6.72
Deeb 2001b	29	8.43	1	59	8.54	1	-0.11 [-0.55, 0.33]	7.49
Tupola 2001	22	-.4	.7	22	.2	.8	-0.60 [-1.04, -0.16]	7.50
Holcombe 2002	457	8.7	1.65	457	8.69	1.52	0.01 [-0.20, 0.22]	34.99
Ford-Adams 2003	12	8.4	1	11	8.9	1	-0.50 [-1.32, 0.32]	2.21
ANA-2126 (Lispro)	48	.1	1	95	-.1	1	0.20 [-0.15, 0.55]	12.29
Heterogeneity: $I^2 = 40.00\%$, $H^2 = 1.67$							-0.07 [-0.20, 0.07]	
Test of $\theta_i = \theta_j$: Q(6) = 10.00, p = 0.12								
Test of θ = 0: z = -0.94, p = 0.35								
**Overall**							-0.05 [-0.17, 0.07]	
Heterogeneity: $I^2 = 21.34\%$, $H^2 = 1.27$								
Test of $\theta_i = \theta_j$: Q(8) = 10.17, p = 0.25								
Test of θ = 0: z = -0.85, p = 0.40								
Test of group differences: $Q_b(1) = 0.17$, p = 0.68								

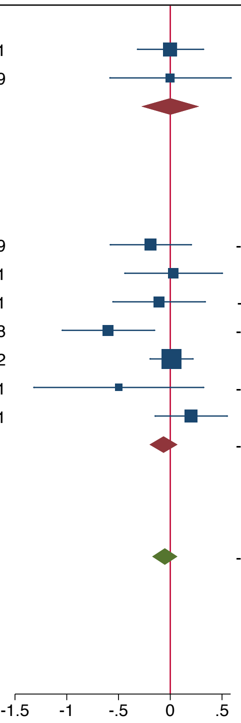

Fixed-effects inverse-variance model

Figure S18: Fixed-effects meta-analysis of regular human insulins (RHI) versus rapid-acting insulin analogues (RAIA) on postprandial glucose level

Study	RHI N	RHI Mean	RHI SD	RAIA N	RAIA Mean	RAIA SD	Mean diff. with 95% CI	Weight (%)
Fairchild 2000	35	11.73	1.04	35	12.14	2.62	-0.41 [-1.34, 0.52]	21.09
Deeb 2001a	30	15	5.4	59	11.7	4.4	3.30 [1.21, 5.39]	4.21
Deeb 2001b	29	15	5.4	59	13.5	5.5	1.50 [-0.93, 3.93]	3.11
Tupola 2001	22	-.9	5.4	22	.3	4.5	-1.20 [-4.14, 1.74]	2.13
Holcombe 2002	457	10.6	4.3	457	9.7	4	0.90 [0.36, 1.44]	63.44
Tubiana-Rufi 2004	27	11.1	6.1	27	11.1	5	0.00 [-2.98, 2.98]	2.08
Danne 2007	23	1.63	3.5	24	2.02	4.02	-0.39 [-2.55, 1.77]	3.95
**Overall**							0.63 [0.20, 1.06]	

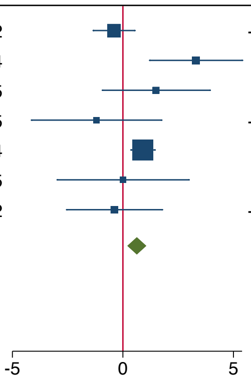

Heterogeneity: I^2 = 60.02%, H^2 = 2.50

Test of $\theta_i = \theta_j$: Q(6) = 15.01, p = 0.02

Test of θ = 0: z = 2.87, p = 0.00

Fixed-effects inverse-variance model

Figure S19: Random-effects meta-analysis of regular human insulins (RHI) versus rapid-acting insulin analogues (RAIA) on postprandial glucose level

Study	RHI N	RHI Mean	RHI SD	RAIA N	RAIA Mean	RAIA SD	Mean diff. with 95% CI	Weight (%)
Fairchild 2000	35	11.73	1.04	35	12.14	2.62	-0.41 [-1.34, 0.52]	23.21
Deeb 2001a	30	15	5.4	59	11.7	4.4	3.30 [1.21, 5.39]	12.23
Deeb 2001b	29	15	5.4	59	13.5	5.5	1.50 [-0.93, 3.93]	10.13
Tupola 2001	22	-.9	5.4	22	.3	4.5	-1.20 [-4.14, 1.74]	7.76
Holcombe 2002	457	10.6	4.3	457	9.7	4	0.90 [0.36, 1.44]	27.29
Tubiana-Rufi 2004	27	11.1	6.1	27	11.1	5	0.00 [-2.98, 2.98]	7.62
Danne 2007	23	1.63	3.5	24	2.02	4.02	-0.39 [-2.55, 1.77]	11.77
**Overall**							0.57 [-0.38, 1.52]	

Heterogeneity: $\tau^2 = 0.79$, $I^2 = 60.02\%$, $H^2 = 2.50$

Test of $\theta_i = \theta_j$: $Q(6) = 15.01$, $p = 0.02$

Test of $\theta = 0$: $z = 1.17$, $p = 0.24$

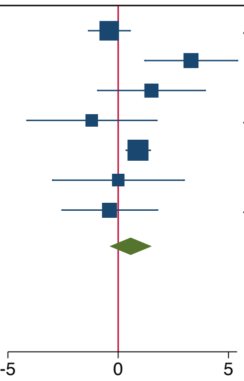

Random-effects DerSimonian–Laird model

Figure S20: Trial Sequential Analysis of regular human insulins (RHI) versus rapid-acting insulin analogues (RAIA) on postprandial glucose level

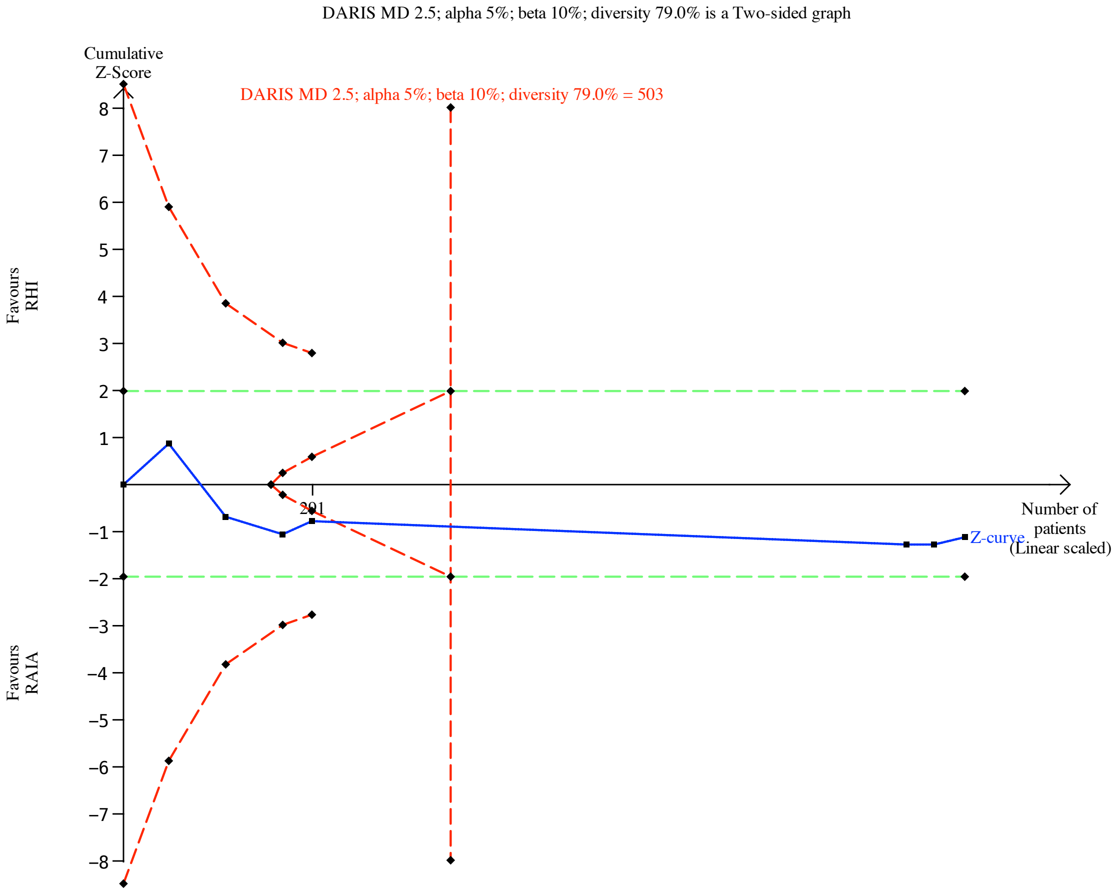

Figure S21: Subgroup analysis on age of regular human insulins (RHI) versus rapid-acting insulin analogues (RAIA) on postprandial glucose level

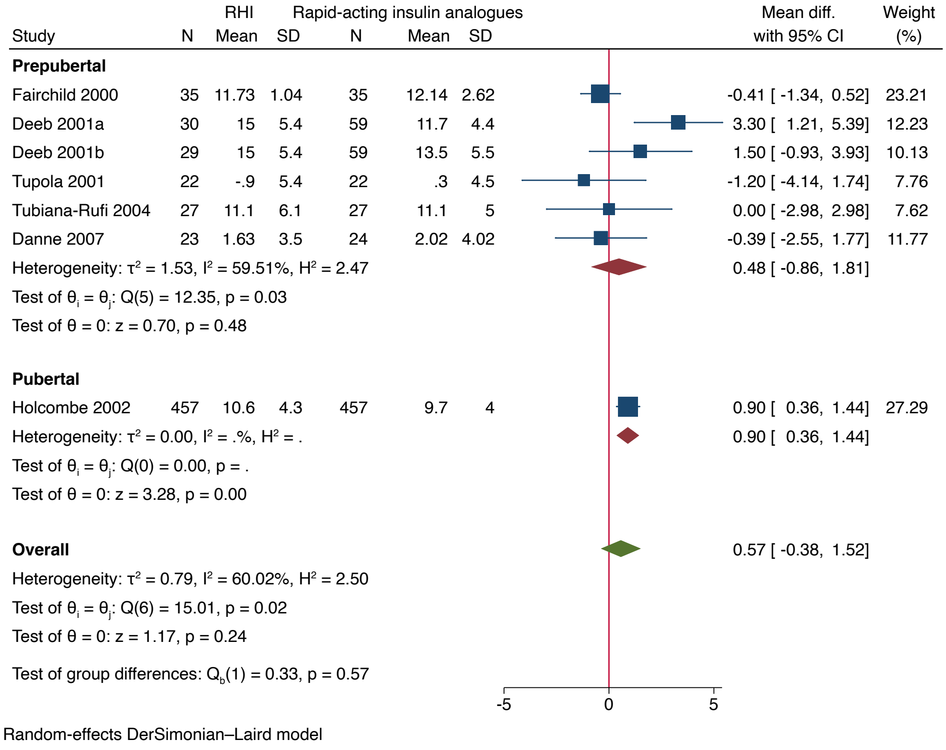

Figure S22: Subgroup analysis on insulin lispro versus insulin aspart of regular human insulins (RHI) versus rapid-acting insulin analogues (RAIA) on postprandial glucose level

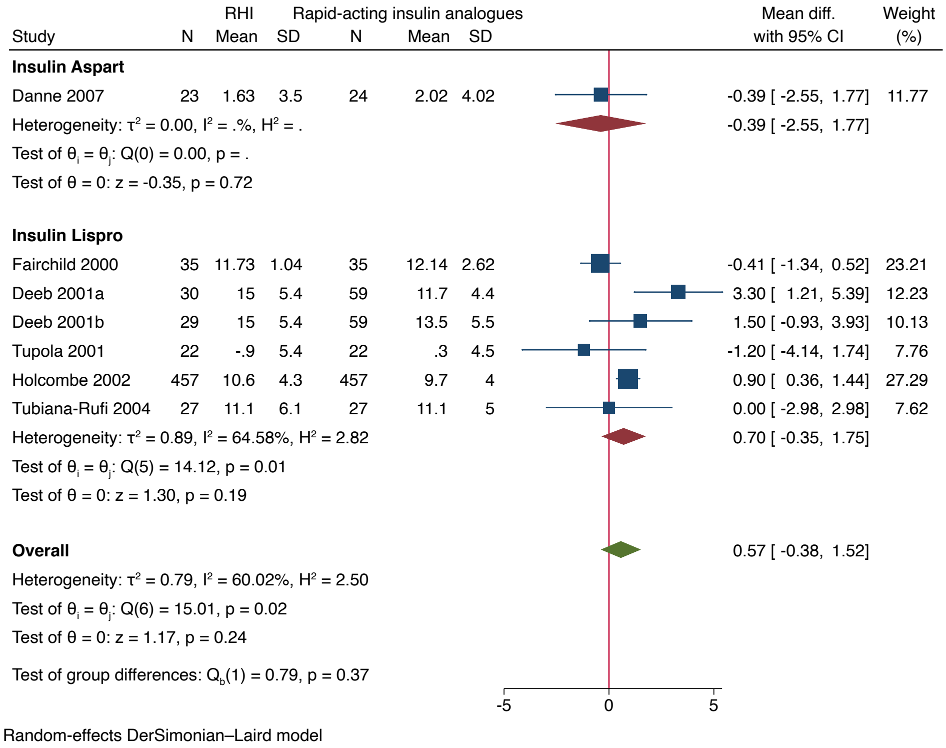

Figure S23: Subgroup analysis on method of delivery of regular human insulins (RHI) versus rapid-acting insulin analogues (RAIA) on postprandial glucose level

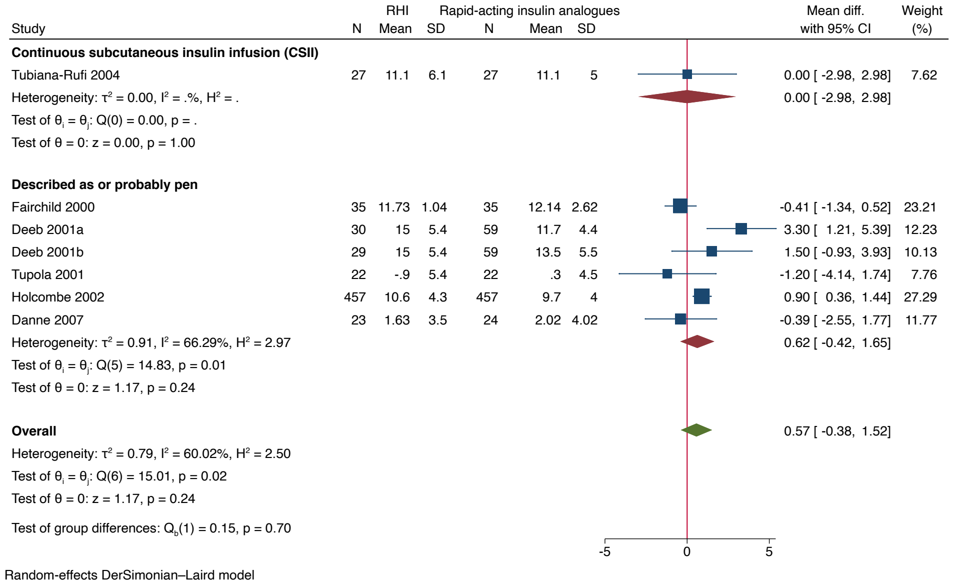

Figure S24: Meta-analysis of regular human insulins (RHI) versus rapid-acting insulin analogues (RAIA) on severe hypoglycemia (parallel trials)

Study	RHI Event	RHI No event	RAIA Event	RAIA No event	Risk ratio with 95% CI	Weight (%)
ANA-2126 (Aspart)	5	43	11	176	1.77 [0.65, 4.85]	39.42
ANA-2126 (Lispro)	4	44	8	87	0.99 [0.31, 3.12]	47.11
Pankowska 2010	0	21	1	19	0.32 [0.01, 7.38]	13.46
**Overall**					1.21 [0.59, 2.48]	

Heterogeneity: $I^2 = 0.00\%$, $H^2 = 1.00$

Test of $\theta_i = \theta_j$: $Q(2) = 1.36$, $p = 0.51$

Test of $\theta = 0$: $z = 0.51$, $p = 0.61$

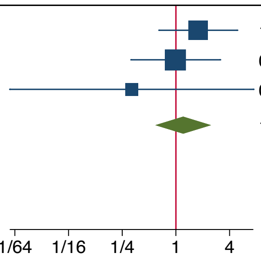

Fixed-effects Mantel–Haenszel model

Figure S25: Meta-analysis of regular human insulins (RHI) versus rapid-acting insulin analogues (RAIA) on HbA1c (parallel trials)

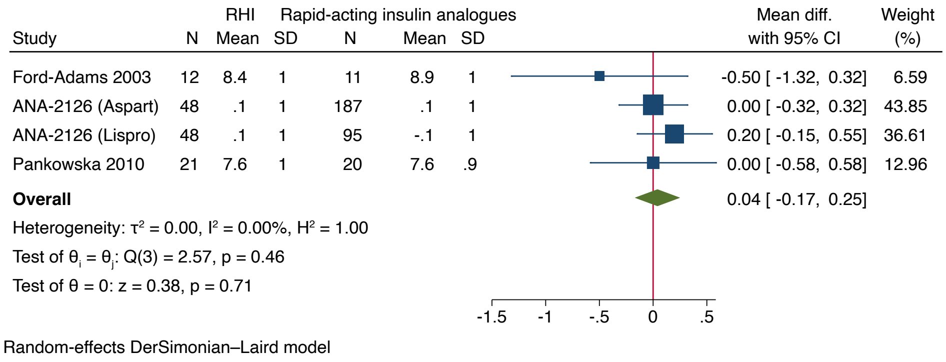

Figure S26: Trial Sequential Analysis of regular human insulins (RHI) versus rapid-acting insulin analogues (RAIA) on HbA1c (parallel trials)

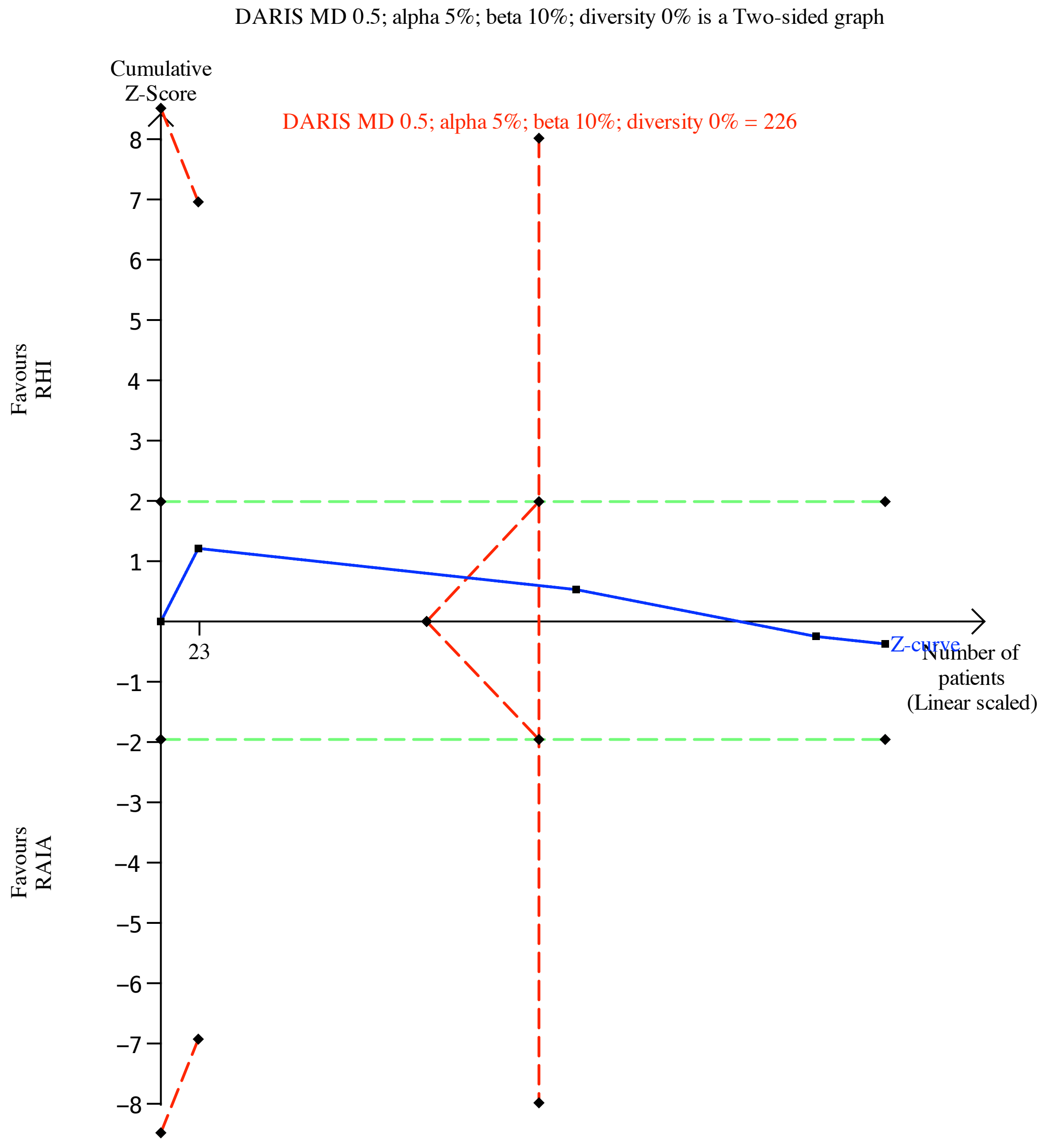

Supplementary tables

Table S1: Characteristics of included trials

Trial-ID	Registry/protocol	Risk of for profit bias	Income setting	Trial design	Age group	Type of control	Administration time RHI	Administration time RAIA	Number randomized
Fairchild 2000 [1]	Not registered	Yes	High-income economies	Crossover	Prepubertal (5-10 years)	Insulin Lispro	30 minutes before meals	Immediately before meals	35
Deeb 2001a [2]	Not registered	Yes	High-income economies	Crossover	Prepubertal (3-12 years)	Insulin Lispro	30-45 minutes before meals	Within 15 minutes before meals	32
Deeb 2001b [2]	Not registered	Yes	High-income economies	Crossover	Prepubertal (3-12 years)	Insulin Lispro	30-45 minutes before meals	Immediately after meals	28
Tupola 2001 [3]	Not registered	Yes	High-income economies	Crossover	Prepubertal (<10 years)	Insulin Lispro	20-30 minutes before meals	Within 30 minutes from the start of the meal	24
Holcombe 2002 [4]	Not registered	Yes	High-income economies	Crossover	Pubertal (9-18 years)	Insulin Lispro	30-45 minutes before meals	Immediately before meals	463
Ford-Adams 2003 [5]	Not registered	Yes	High-income economies	Crossover	Prepubertal (7-11 years)	Insulin Lispro	20-30 minutes before meals	Immediately before meals	23
Tubiana-Rufi 2004 [6]	Not registered	Yes	High-income economies	Crossover	Prepubertal	Insulin Lispro	20-30 minutes before meals	0–5 minutes before meals	27
Cherubini 2006 [7]	Not registered	Yes	High-income economies	Paralllel	Prepubertal (7-11 years)	Insulin Aspart	30 minutes before meals	2 minutes before meals	30

ANA-2126 (Aspart) [8]	NCT00071448	Yes	High-income economies	Paralllel	Prepubertal and pubertal (6-18 years)	Insulin Aspart	20-30 minutes before meals	Immediately before meals	235
ANA-2126 (Lispro) [8]	NCT00071448	Yes	High-income economies	Paralllel	Prepubertal and pubertal (6-18 years)	Insulin Lispro	20-30 minutes before meals	Immediately before meals	143
Danne 2007 [9]	NCT01467141	Yes	High-income economies	Crossover	Prepubertal (2-6 years)	Insulin Aspart	30 minutes before meals	Shortly before or after meals	26
Pankowska 2010 [10]	NCT00571935	Yes	High-income economies	Paralllel	Prepubertal (<7 years)	Insulin Aspart	15-30 minutes before meals	Immediately before meals	41
**RHI**: Regular human insulins. **RAIA**: Rapid-acting insulin analogues.									

1. Fairchild JM, Ambler GR, Genoud-Lawton CH, Westman EA, Chan A, Howard NJ, et al. Insulin lispro versus regular insulin in children with type 1 diabetes on twice daily insulin. Pediatr Diabetes. 2000;1(3):135-41.
2. Deeb LC, Holcombe JH, Brunelle R, Zalani S, Brink S, Jenner M, et al. Insulin lispro lowers postprandial glucose in prepubertal children with diabetes. Pediatrics. 2001;108(5):1175-9.
3. Tupola S, Komulainen J, Jaaskelainen J, Sipila I. Post-prandial insulin lispro vs. human regular insulin in prepubertal children with Type 1 diabetes mellitus. Diabet Med. 2001;18(8):654-8.
4. Holcombe JH, Zalani S, Arora VK, Mast CJ, Lispro in Adolescents Study G. Comparison of insulin lispro with regular human insulin for the treatment of type 1 diabetes in adolescents. Clin Ther. 2002;24(4):629-38.
5. Ford-Adams ME, Murphy NP, Moore EJ, Edge JA, Ong KL, Watts AP, et al. Insulin lispro: a potential role in preventing nocturnal hypoglycaemia in young children with diabetes mellitus. Diabet Med. 2003;20(8):656-60.
6. Tubiana-Rufi N, Coutant R, Bloch J, Munz-Licha G, Delcroix C, Montaud-Raguideau N, et al. Special management of insulin lispro in continuous subcutaneous insulin infusion in young diabetic children: a randomized cross-over study. Horm Res. 2004;62(6):265-71.
7. Cherubini V, Iannilli A, Iafusco D, Cardella F, Giamprini MS, Fanelli C, Coppa GV. Premeal insulin treatment during basal-bolus regimen in young children with type 1 diabetes. Diabetes Care. 2006;29(10):2311-2.
8. ANA-2126. Basal/Bolus Therapy with Insulin Aspart (NovoLog®) versus Regular Human Insulin (Novolin®R) or Insulin Lispro (Humalog®) in Combination with NPH: An Open-Label, Randomized, Parallel-Group, Multicenter Study in Children and Adolescents with Type 1 Diabetes. Novo Nordisk. 2006.

9. Danne T, Rastam J, Odendahl R, Nake A, Schimmel U, Szczepanski R, et al. Parental preference of prandial insulin aspart compared with preprandial human insulin in a basal-bolus scheme with NPH insulin in a 12-wk crossover study of preschool children with type 1 diabetes. Pediatr Diabetes. 2007;8(5):278-85.
10. Pankowska E, Nazim J, Szalecki M, Urban M. Equal metabolic control but superior caregiver treatment satisfaction with insulin aspart in preschool children. Diabetes Technol Ther. 2010;12(5):413-8.

Table S2: Risk of bias

Study	Risk of bias domains: D1	D2	D3	D4	D5	Overall
Fairchild 2000 - Severe hypoglycemia	-	X	X	X	-	X
Fairchild 2000 - HbA1c	-	X	X	+	-	X
Fairchild 2000 - Postprandial blood glucose level	-	X	X	+	-	X
Fairchild 2000 - Serious adverse events	-	X	X	X	-	X
Fairchild 2000 - Quality of life (child)	-	X	X	X	-	X
Fairchild 2000 - Non-serious adverse events	-	X	X	X	-	X
Fairchild 2000 - Quality of life (parents/caregivers)	-	X	X	X	-	X
Deeb 2001 - Severe hypoglycemia	-	X	+	X	-	X
Deeb 2001 - HbA1c	-	X	+	+	-	X
Deeb 2001 - Postprandial glucose level	-	X	+	+	-	X
Deeb 2001 - Serious adverse events	-	X	+	X	-	X
Deeb 2001 - Non-serious adverse events	-	X	+	X	-	X
Deeb 2001 - Nocturnal hypoglycemia	-	X	+	X	-	X
Tupola 2001 - Severe hypoglycemia	-	-	X	X	-	X
Tupola 2001 - HbA1c	-	-	X	+	-	X
Tupola 2001 - Postprandial glucose level	-	-	X	+	-	X
Tupola 2001 - Quality of life (child)	-	-	X	X	-	X
Tupola 2001 - Height	-	-	X	X	-	X
Tupola 2001 - Weight	-	-	X	X	-	X
Tupola 2001 - Quality of life (parents/caregivers)	-	-	X	X	-	X
Holcombe 2002 - Severe hypoglycemia	-	-	+	X	-	X
Holcombe 2002 - HbA1c	-	-	+	+	-	X
Holcombe 2002 - Postprandial glucose level	-	-	+	+	-	X
Holcombe 2002 - Serious adverse events	-	-	+	X	-	X
Holcombe 2002 - Non-serious adverse events	-	-	+	X	-	X
Ford-Adams 2003 - Severe hypoglycemia	+	X	+	X	-	X
Ford-Adams 2003 - HbA1c	+	X	+	+	-	X
Tubiana-Rufi 2004 - Severe hypoglycemia	-	X	X	X	-	X
Tubiana-Rufi 2004 - Ketoacidosis	-	X	X	X	-	X
Tubiana-Rufi 2004 - HbA1c	-	X	X	+	-	X
Tubiana-Rufi 2004 - Postprandial glucose level	-	X	X	+	-	X
Tubiana-Rufi 2004 - Quality of life (parents/caregivers)	-	X	X	+	-	X
Tubiana-Rufi 2004 - Nocturnal hypoglycemia	-	X	X	+	-	X
ANA-2126 - Severe hypoglycemia	-	X	X	X	-	X
ANA-2126 - Ketoacidosis	-	X	X	X	-	X
ANA-2126 - Serious adverse events	-	X	X	X	-	X
ANA-2126 - HbA1c	-	X	X	+	-	X
ANA-2126 - Non-serious adverse events	-	X	X	X	-	X
ANA-2126 - Height	-	X	X	X	-	X
ANA-2126 - Weight	-	X	X	X	-	X
Danne 2007 - Severe hypoglycemia	-	X	X	X	-	X
Danne 2007 - Postprandial glucose level	-	X	X	+	-	X
Danne 2007 - Serious adverse events	-	X	X	X	-	X
Danne 2007 - HbA1c	-	X	X	+	-	X
Danne 2007 - Non-serious adverse events	-	X	X	X	-	X
Danne 2007 - Quality of life (parents/caregiver)	-	X	X	X	-	X
Pankowska 2010 - Severe hypoglycemia	-	X	+	X	-	X
Pankowska 2010 - Serious adverse events	-	X	+	X	-	X
Pankowska 2010 - HbA1c	-	X	+	+	-	X
Pankowska 2010 - Non-serious adverse events	-	X	+	X	-	X
Pankowska 2010 - All-cause mortality	-	X	+	+	-	X
Pankowska 2010 - Quality of life (child)	-	X	+	X	-	X
Pankowska 2010 - Quality of life (parents/caregiver)	-	X	+	X	-	X
Cherubini 2006 - HbA1c	-	X	X	X	-	X
Cherubini 2006 - Postprandial glucose level	-	X	X	X	-	X

Domains:
D1: Bias arising from the randomization process.
D2: Bias due to deviations from intended intervention.
D3: Bias due to missing outcome data.
D4: Bias in measurement of the outcome.
D5: Bias in selection of the reported result.

Judgement
X High
- Some concerns
+ Low

Table S3: Trial definitions of outcomes

Severe hypoglycemia	
Fairchild 2000	*Hypoglycemic episodes with convulsion or coma.*
Deeb 2001	*Hypoglycemic episodes needing the assistance of others, resulting in coma, or requiring intravenous glucose or glucagon.*
Tupola 2001	*Hypoglycemic episodes resulting in unconsciousness.*
Holcombe 2002	*Hypoglycemic episodes needing the assistance of others or for intravenous glucose or glucagon injection to treat hypoglycemia.*
Ford-Adams 2003	*Hypoglycemic episodes with convulsions or requiring glucagon administration.*
Tubiana-Rufia 2004	No description.
Cherubini 2006	*Hypoglycemic episodes with seizures or a clinical condition in which the child is unconscious.*
ANA-2126	No description.
Danne 2007	*Hypoglycemic episodes needing the assistance of others.*
Pankowska 2010	*Hypoglycemic episodes with blood glucose <2.8 mmol/L (50 mg/dL) accompanied by severe central nervous system symptoms consistent with hypoglycemia and needing the assistance of others.*
Ketoacidosis	
Tubiana-Rufia 2004	No description.
ANA-2126	No description.
Serious adverse events	
Fairchild 2000	No description.
Deeb 2001	No description.
Holcombe 2002	No description.
ANA-2126	No description.
Danne 2007	No description.
Pankowska 2010	*AEs were defined as serious (SAEs) if they resulted in persistent, significant disability, hospitalization, a life-threatening event, or death.*
Quality of life (child)	
Fairchild 2000	*At the conclusion of the study a patient/parent preference questionnaire was administered.*
Tupola 2001	No description.
Pankowska 2010	No description.
HbA1c	
Fairchild 2000	*HbA1c was measured at a central laboratory using the Bio-Rad Diamat analyser, (Hercules, CA).*
Deeb 2001	*HbA1c levels were determined using high performance liquid chromatogr phy at a central laboratory (Covance Laboratories, Indianapolis, IN; reference range: 4.3%–6.1%).*
Tupola 2001	*HbA1c (high pressure liquid chromatography, reference limit 4± 6%) was measured (...) at each visit.*
Holcombe 2002	*In the present study, HbA1c assays were performed by a central laboratory (Covance, Indianapolis, Ind; reference range, 4.3%-6.1%) using ion-exchange high-performance liquid chromatography (Bio-Rad Variant Analyzer, Clinical Diagnostics, Hercules, Calif).*
Ford-Adams 2003	*HbA1c was measured by high-pressure liquid chromatography (HPLC); interassay CVs were 1.3% at 5.4%, and 1.1% at 9.6%.*

Tubiana-Rufia 2004	*HbA1c evaluation was centralized – high performance liquid chromatography on a variant I system program A1c (Bio-Rad, Richmond, Calif., USA) certified to the DCCT reference method (mean normal reference B 2 SD = 5.1 B 0.6%).*
Cherubini 2006	No description.
ANA-2126	No description.
Danne 2007	No description
Pankowska 2010	*A1C was determined centrally at a certified laboratory during screening and randomization and at the beginning and end of active treatment. A1C was assayed using high-performance liquid chromatography (Variant, Bio-Rad, Hercules, CA) with a reference interval of 4.7–6.4% hemoglobin.*
Non-serious adverse events	
Fairchild 2000	No description.
Deeb 2001	No description.
Holcombe 2002	No description.
ANA-2126	No description.
Danne 2007	No description.
Pankowska 2010	*AEs were mild if they caused no interruption in daily activities.*
All-cause mortality	
Pankowska 2010	No definition.
Postprandial glucose level	
Fairchild 2000	No description.
Deeb 2001	*OneTouch blood glucose meters (Lifescan, Inc, Milpitas, CA) were provided by the investigator.*
Tupola 2001	*They measured seven-point glucose profiles (before breakfast, 1 h and 2 h after breakfast, before dinner, 1 h and 2 h after dinner, and at bedtime) at home 1 day during the week before each visit.*
Holcombe 2002	*Postprandial glucose level was measured 2-hour postprandial.*
Tubiana-Rufia 2004	*Postprandial BG excursions, defined as a change in glucose concentration from before to 2 h after a meal, were calculated for each patient and for each meal.*
Cherubini 2006	*During the 18-week study period, parents were asked to perform five blood glucose tests everyday on their children: fasting (6:00 – 9:00 P.M.), before meal (breakfast, lunch, afternoon snack, and supper), and at bedtime (11:00 P.M.). Twice a week, selfblood glucose monitoring was also requested at 2:00 A.M. and at 10:00 A.M*
Danne 2007	*From the 7-point blood glucose (BG) profiles recorded at baseline (Visit 2) end of first treatment period (Visit 5) and end of second treatment period (Visit 8), the postprandial incremental blood glucose was derived as the average of post-meal BG minus pre-meal BG measurements over all three daily meals.*
Continuous blood glucose monitoring	
Pankowska 2010	*The difference between maximum and minimum glucose levels as measured by CGMS over a 24-h period.*
Changes in height	
Tupola 2001	*Patients' height, weight, and insulin dose were recorded at each visit.*
Changes in weight	
Tupola 2001	*Patients' height, weight, and insulin dose were recorded at each visit.*
ANA-2126	No description.
Quality of life (caregiver)	
Fairchild 2000	*At the conclusion of the study a patient/parent preference questionnaire was administered.*
Tupola 2001	No description.
Tubiana-Rufia 2004	*Parents' and children's satisfaction with each treatment was assessed by questionnaires filed in by the parents at the end of each period of treatment, and*

	preference for one insulin or the other was assessed by questionnaires filed in by the parents at the end of the study.
Danne 2007	*Treatment satisfaction was evaluated using a modified version of the WHO Diabetes Treatment Satisfaction Questionnaire (DTSQ-WHO); the modified questionnaire (DTSQ-M) is adapted for the subject's parents/legal representatives (referred to as parents in this article) but is otherwise very similar to the standard version. The modified version has previously been used in trials with preschool children (6); however, in contrast to DTSQ-WHO, the DTSQ-M is not a validated questionnaire. In the DTSQ-M, the parents are answering seven questions regarding their child's status. Five of the questions are almost identical to those used in DTSQWHO; but three questions in the DTSQ-WHO related to convenience, flexibility, and treatment satisfaction are replaced by two questions related to general health and interference with school activities of the children in the DTSQ-M.*
Pankowska 2010	No description.
Nocturnal hypoglycaemia	
Fairchild 2000	*A hypoglycaemic episode was defined as any time a patient felt (or another person observed) that he or she was experiencing a sign/symptom that would be associated with hypoglycaemia (where possible confirmed by a blood glucose level <4.0 mmol/L) or any asymptomatic blood glucose measurement less than 3.0 mmol/L.*
Deeb 2001	*Hypoglycemia was defined as any time a patient had symptoms associated with hypoglycemia or had measured blood glucose <3.5 mmol/L.*
Holcombe 2002	*A hypoglycemic episode was defined as any occasion on which a patient experienced (or was observed to have) symptoms associated with hypoglycemia, or on which the patient had a measured BG level ~3.0 mmol/L.*
Ford-Adams 2003	*Data from overnight metabolic profiles were divided into three time periods: period 1, 18.00–22.00 h (post-evening meal to bedtime); period 2, 22.00–04.00 h (early night); period 3, 04.00–07.00 h (early morning). Prevalence of low blood glucose levels was calculated as the proportion of total measurements < 3.5 mmol/l in each time period.*
Tubiana-Rufia 2004	No description.
ANA-2126	*Nocturnal minor hypoglycemia (23:00 to 6:00).*

Table S4: Trial results not suitable for meta-analyses

Serious adverse events	
Fairchild 2000	*No other adverse events* (besides hypoglycaemia) *could be attributed to insulin lispro, however, one boy, with a past history of lipoatrophy on other insulins, developed lipoatrophy at the end of his 3 months on insulin lispro.*
Deeb 2001	*With respect to other adverse events, no significant differences were observed among the 3 therapies. The most common adverse events were rhinitis, pharyngitis, cough, and fever, none of which showed a relationship to the insulin therapies.*
Holcombe 2002	*No differences were observed between treatments with respect to other adverse events, the most common of which were rhinitis, pharyngitis, and flu syndrome.*
Danne 2007	*Adverse events were few, and the majority were mild. Upper respiratory tract infections, gastroenteritis, cough, bronchitis and pyrexia were the most frequent events. Three serious adverse events, a hand fracture, a hypoglycaemic episode and a severe gastroenteritis were recorded, none of which were considered related to the treatment.*
Quality of life (child)	
Fairchild 2000	*The majority of families (28/35) preferred using insulin lispro because of its greater convenience, with 25 (71%) continuing on insulin lispro after the study. The seven children whose families chose to use regular insulin after the study were doing so because of better glycaemic control or the perception that their blood glucose profile was more stable.*
Tupola 2001	*After the study, 18/22 (82%) patients and their families wanted to continue treatment with pre- or post-prandial insulin lispro because of its convenience.*
Pankowska 2010	*There was a statistically significant difference in QoL total scores between the treatment groups. For self-reported hypoglycaemic episodes, there was an improvement observed in the NovoRapid® CSII group and a statistically significant difference between the treatment groups. However, no statistically significant difference was observed between the treatment groups in self-reported hyperglycaemia.*<br><br>NB: Allocation to insulin aspart CSII is not randomized and thus not included.
HbA1c	
Tubiana-Rufia 2004	*Changes in HbA1c levels did not significantly differ between the treatments: +0.15 +/- 0.13% (*insulin lispro*) vs. +0.11 +/- 0.63% (*regular human insulins*).*
Cherubini 2006	*A1C decreased in group A (*regular human insulins*, from 7.5 +/- 0.8 to 7.0 +/- 0.4%) but not in group B (*insulin aspart*, from 7.5 +/- 1.4 to 7.4 +/- 0.5%) (p=0.018).*
Danne 2007	*The HbA1c and fructosamine levels remained stable throughout the trial: at the end of trial, HbA1c ranged from 7.6 to 7.7% and fructosamine from 299 to 301 mmol/L, and no significant difference between treatments was found. Throughout the trial, the mean values collected for HbA1c, fructosamine, and seven-point BG (not shown) showed a consistent pattern and documented that the glycemic control achieved with the two treatments was equal and the values were either constant or – at least for fructosamine – improved slightly during the trial.*
Non-serious adverse events	
Fairchild 2000	*No other adverse events (*besides hypoglycaemia) *could be attributed to insulin lispro, however, one boy, with a past history of lipoatrophy on other insulins, developed lipoatrophy at the end of his 3 months on insulin lispro.*

Deeb 2001	*With respect to other adverse events, no significant differences were observed among the 3 therapies. The most common adverse events were rhinitis, pharyngitis, cough, and fever, none of which showed a relationship to the insulin therapies.*
Holcombe 2002	*No differences were observed between treatments with respect to other adverse events, the most common of which were rhinitis, pharyngitis, and flu syndrome.*
ANA-2126	*The number and types of treatment emergent adverse events (TEAEs) that occurred in this study were not uncommon for the subject population enrolled in the study. No notable differences in the occurrence of adverse events were observed between treatments*
Danne 2007	*Adverse events were few, and the majority were mild. Upper respiratory tract infections, gastroenteritis, cough, bronchitis and pyrexia were the most frequent events.*
Postprandial glucose level	
Cherubini 2006	*2-h postprandial blood glucose values were (...) similar (143 +/- 19 vs. 133 +/- 18, group A (*regular human insulins*) vs. B* (insulin aspart)*, respectively; p=0.175).*
Changes in height	
Tupola 2001	*Nor were there differences in weight for height.*
ANA-2126	*Treatment with insulin aspart +NPH, Novolin R+NPH, or lispro+NPH did not have an adverse effect upon physical examination findings, vital signs, weight, or hematology, blood chemistry, or lipid laboratory values.*
Changes in weight	
Tupola 2001	*(...) nor were there differences in weight for height.*
ANA-2126	*Treatment with insulin aspart +NPH, Novolin R+NPH, or lispro+NPH did not have an adverse effect upon physical examination findings, vital signs, weight, or hematology, blood chemistry, or lipid laboratory values.*
Quality of life (caregiver)	
Fairchild 2000	*The majority of families (28/35) preferred using insulin lispro because of its greater convenience, with 25 (71%) continuing on insulin lispro after the study. The seven children whose families chose to use regular insulin after the study were doing so because of better glycaemic control or the perception that their blood glucose profile was more stable.*
Tupola 2001	*After the study, 18/22 (82%) patients and their families wanted to continue treatment with pre- or post-prandial insulin lispro because of its convenience.*
Tubiana-Rufia 2004	*The quality of metabolic control perceived by the parents did not significantly differ between the 2 insulin treatments. Parents reported that their own and the child's daily life was easier with LP by comparison with RH (70 vs. 26%, p = 0.02). The main reasons were the following: less constraints for timing of the meals (no delayed meals after boluses, 74 vs. 11%, p = 0.01), and easier management with LP of special age-related difficulties such as acute infections, unpredict- able behavior patterns, or day-to-day variations in diet and physical activities (73 vs. 12%, p = 0.01). At the end of the study, their choice was to continue treatment with LP in 74% of the cases (LP vs. RH, p = 0.01).*
Danne 2007	*Except for a question about frequency of low blood sugar (question 3), the mean score difference (IAsp–HI) was positive for all the DTSQ-M questions. The difference was statistically significant (p=0.045) for question 7, "How satisfied would you be to continue your child's present form of insulin?", and almost significant (p=0.051) for question 6, "Would you recommend the form of insulin treatment your child receives to someone else?". A post hoc analysis (not shown) indicates that the order in which IAsp and HI was scored might have an impact on the change in scores.*

Pankowska 2010	*Questionnaire results for caregivers were subdivided into treatment satisfaction total score and subscores for satisfaction with the frequency of hypoglycemia and hyperglycemia. IAsp CSII and IAsp MDI demonstrated a significant increase in treatment satisfaction during the trial, but the increase in mean treatment satisfaction total score was significantly greater for CSII compared to the IAsp and HI MDI groups and significantly greater for IAsp MDI compared with HI MDI (P=0.04 among groups). Caregivers of children receiving IAsp CSII found the frequency of self-reported hypoglycemic events during treatment acceptable (score increased by 0.9 points), whereas the decrease in scores among caregivers of children in the IAsp and HI MDI groups (-1.1 and -0.6 points, respectively; P=0.0002 vs. CSII) suggested an increase in the perception of hypoglycemic episodes.*<br><br>NB: Allocation to insulin aspart CSII is not randomized and thus not included.
Nocturnal hypoglycaemia	
Deeb 2001	*No significant difference in the number of hypoglycemic episodes by time-of-day was observed among the 3 therapies.*
Tubiana-Rufia 2004	*The mean nocturnal basal rate was similar.*

Table S5: Summary of findings table

Summary of findings:

Regular human insulins compared to rapid-acting insulin analogues for type 1 diabetes in children and adolescents

Patient or population: type 1 diabetes in children and adolescents
Setting:
Intervention: regular human insulins
Comparison: rapid-acting insulin analogues

Outcomes	Anticipated absolute effects* (95% CI)		Relative effect (95% CI)	№ of participants (studies)	Certainty of the evidence (GRADE)	Comments
	Risk with rapid-acting insulin analogues	Risk with regular human insulins				
Severe hypoglycemia follow-up: range 3 months to 26 weeks	39 per 1.000	**50 per 1.000** (31 to 79)	**RR 1.28** (0.81 to 2.03)	1769 (9 RCTs)	⨁◯◯◯ Very low[a,b,c]	
Ketoacidosis follow-up: range 16 weeks to 26 weeks	39 per 1.000	**34 per 1.000** (10 to 114)	**RR 0.88** (0.26 to 2.93)	432 (2 RCTs)	⨁◯◯◯ Very low[c,d]	
Serious adverse events follow-up: range 24 weeks to 26 weeks	70 per 1.000	**70 per 1.000** (31 to 156)	**RR 1.00** (0.44 to 2.25)	419 (2 RCTs)	⨁◯◯◯ Very low[c,d]	
HbA1c follow-up: range 3 months to 26 weeks		MD **0.05 lower** (0.17 lower to 0.07 higher)	-	1647 (7 RCTs)	⨁◯◯◯ Very low[e,f]	
Non-serious adverse events follow-up: 26 weeks	500 per 1.000		not estimable	41 (1 RCT)	⨁◯◯◯ Very low[c,g]	
All-cause mortality follow-up: 26 weeks	0 per 1.000		not estimable	42 (1 RCT)	⨁◯◯◯ Very low[c,g]	
Postprandial glucose level follow-up: range 3 months to 16 weeks		MD **0.57 higher** (0.38 lower to 1.52 higher)	-	1306 (6 RCTs)	⨁◯◯◯ Very low[h,i]	
Nocturnal hypoglycemia follow-up: 24 weeks	411 per 1.000		not estimable	378 (1 RCT)	⨁◯◯◯ Very low[c,g]	

***The risk in the intervention group** (and its 95% confidence interval) is based on the assumed risk in the comparison group and the **relative effect** of the intervention (and its 95% CI).

CI: confidence interval; **MD:** mean difference; **RR:** risk ratio

GRADE Working Group grades of evidence
High certainty: we are very confident that the true effect lies close to that of the estimate of the effect.
Moderate certainty: we are moderately confident in the effect estimate: the true effect is likely to be close to the estimate of the effect, but there is a possibility that it is substantially different.
Low certainty: our confidence in the effect estimate is limited: the true effect may be substantially different from the estimate of the effect.
Very low certainty: we have very little confidence in the effect estimate: the true effect is likely to be substantially different from the estimate of effect.

Explanations

a. Downgraded 2 for high risk of bias. All nine included trials had overall high risk of bias and with several domains at high risk of bias.
b. Downgraded 1 level for indirectness in participants. All nine trials were conducted in countries with high-income economies.
c. Downgraded 2 levels for imprecision due to Trial Sequential Analysis showing that there was not enough information to confirm or reject a relative risk reduction (RRR) of 20% and the accrued number of participants is below 5% of the diversity-adjusted required information size (DARIS).
d. Downgraded 2 for high risk of bias. Both included trials had overall high risk of bias and with several domains at high risk of bias.
e. Downgraded 2 for high risk of bias. All seven included trials had overall high risk of bias and with several domains at high risk of bias.
f. Downgraded 1 level for indirectness in participants. All seven trials were conducted in countries with high-income economies.
g. Downgraded 2 for high risk of bias. Based on one trial with overall high risk of bias and with several domains at high risk of bias.

h. Downgraded 2 for high risk of bias. All six included trials had overall high risk of bias and with several domains at high risk of bias.
i. Downgraded 1 level for indirectness in participants. All six trials were conducted in countries with high-income economies.

NB: For short-term outcomes (severe hypoglycaemia, ketoacidosis, HbA1c, postprandial glucose level, and nocturnal hypoglycaemia), each participant provided data in both the intervention and control group, if only data from the end of the trial after crossover were reported. Therefore, the number analysed per outcome may be higher than the total number randomised.

Supplementary text

Text S1: PRISMA checklist

Section and Topic	Item #	Checklist item	Location where item is reported
**TITLE**			
Title	1	Identify the report as a systematic review.	Title (page 1)
**ABSTRACT**			
Abstract	2	See the PRISMA 2020 for Abstracts checklist.	
**INTRODUCTION**			
Rationale	3	Describe the rationale for the review in the context of existing knowledge.	"Introduction" (page 4)
Objectives	4	Provide an explicit statement of the objective(s) or question(s) the review addresses.	"Introduction" (page 4)
**METHODS**			
Eligibility criteria	5	Specify the inclusion and exclusion criteria for the review and how studies were grouped for the syntheses.	"Methods" (page 5)
Information sources	6	Specify all databases, registers, websites, organisations, reference lists and other sources searched or consulted to identify studies. Specify the date when each source was last searched or consulted.	"Methods" (page 5)
Search strategy	7	Present the full search strategies for all databases, registers and websites, including any filters and limits used.	Supplementary text 2 (Text S2)
Selection process	8	Specify the methods used to decide whether a study met the inclusion criteria of the review, including how many reviewers screened each record and each report retrieved, whether they worked independently, and if applicable, details of automation tools used in the process.	"Methods" (page 5)
Data collection process	9	Specify the methods used to collect data from reports, including how many reviewers collected data from each report, whether they worked independently, any processes for obtaining or confirming data from study investigators, and if applicable, details of automation tools used in the process.	"Methods" (page 5)
Data items	10a	List and define all outcomes for which data were sought. Specify whether all results that were compatible with each outcome domain in each study were sought (e.g. for all measures, time points, analyses), and if not, the methods used to decide which results to collect.	"Outcomes and subgroup analyses" (page 5-6)
	10b	List and define all other variables for which data were sought (e.g. participant and intervention characteristics, funding sources). Describe any assumptions made about any missing or unclear information.	"Outcomes and subgroup analyses" (page 5-6)
Study risk of bias assessment	11	Specify the methods used to assess risk of bias in the included studies, including details of the tool(s) used, how many reviewers assessed each study and whether they worked independently, and if applicable, details of automation tools used in the process.	"Methods" (page 5)
Effect measures	12	Specify for each outcome the effect measure(s) (e.g. risk ratio, mean difference) used in the synthesis or presentation of results.	"Assessment of statistical and clinical significance"

Section and Topic	Item #	Checklist item	Location where item is reported
			(page 6)
Synthesis methods	13a	Describe the processes used to decide which studies were eligible for each synthesis (e.g. tabulating the study intervention characteristics and comparing against the planned groups for each synthesis (item #5)).	"Assessment of statistical and clinical significance" (page 6)
	13b	Describe any methods required to prepare the data for presentation or synthesis, such as handling of missing summary statistics, or data conversions.	"Assessment of statistical and clinical significance" (page 6)
	13c	Describe any methods used to tabulate or visually display results of individual studies and syntheses.	"Assessment of statistical and clinical significance" (page 6)
	13d	Describe any methods used to synthesize results and provide a rationale for the choice(s). If meta-analysis was performed, describe the model(s), method(s) to identify the presence and extent of statistical heterogeneity, and software package(s) used.	"Assessment of statistical and clinical significance" (page 6)
	13e	Describe any methods used to explore possible causes of heterogeneity among study results (e.g. subgroup analysis, meta-regression).	"Assessment of statistical and clinical significance" (page 6)
	13f	Describe any sensitivity analyses conducted to assess robustness of the synthesized results.	"Assessment of statistical and clinical significance" (page 6)
Reporting bias assessment	14	Describe any methods used to assess risk of bias due to missing results in a synthesis (arising from reporting biases).	"Assessment of statistical and clinical significance" (page 6)
Certainty assessment	15	Describe any methods used to assess certainty (or confidence) in the body of evidence for an outcome.	"Assessment of statistical and clinical significance"

Section and Topic	Item #	Checklist item	Location where item is reported
			(page 6)
**RESULTS**			
Study selection	16a	Describe the results of the search and selection process, from the number of records identified in the search to the number of studies included in the review, ideally using a flow diagram.	“Results” (page 7)
	16b	Cite studies that might appear to meet the inclusion criteria, but which were excluded, and explain why they were excluded.	“Results” (page 7)
Study characteristics	17	Cite each included study and present its characteristics.	“Results” and “Table of characteristics” (page 7 and table S1)
Risk of bias in studies	18	Present assessments of risk of bias for each included study.	“Results” and “Risk of bias” (page 7 and Table S2)
Results of individual studies	19	For all outcomes, present, for each study: (a) summary statistics for each group (where appropriate) and (b) an effect estimate and its precision (e.g. confidence/credible interval), ideally using structured tables or plots.	“Results” (page 7-10)
Results of syntheses	20a	For each synthesis, briefly summarise the characteristics and risk of bias among contributing studies.	“Results” (page 7-10)
	20b	Present results of all statistical syntheses conducted. If meta-analysis was done, present for each the summary estimate and its precision (e.g. confidence/credible interval) and measures of statistical heterogeneity. If comparing groups, describe the direction of the effect.	“Results” (page 7-10)
	20c	Present results of all investigations of possible causes of heterogeneity among study results.	“Results” (page 7-10)
	20d	Present results of all sensitivity analyses conducted to assess the robustness of the synthesized results.	“Results” (page 7-10)
Reporting biases	21	Present assessments of risk of bias due to missing results (arising from reporting biases) for each synthesis assessed.	“Results” (page 7-10)
Certainty of evidence	22	Present assessments of certainty (or confidence) in the body of evidence for each outcome assessed.	“Results” and “Table S3” (page 7-10)
**DISCUSSION**			
Discussion	23a	Provide a general interpretation of the results in the context of other evidence.	“Discussion” (page 10-13)
	23b	Discuss any limitations of the evidence included in the review.	“Discussion” (page 10-13)
	23c	Discuss any limitations of the review processes used.	“Discussion” (page 10-13)

Section and Topic	Item #	Checklist item	Location where item is reported
	23d	Discuss implications of the results for practice, policy, and future research.	“Discussion” (page 10-13)
**OTHER INFORMATION**			
Registration and protocol	24a	Provide registration information for the review, including register name and registration number, or state that the review was not registered.	“Abstract” (page 3)
	24b	Indicate where the review protocol can be accessed, or state that a protocol was not prepared.	“Methods” (page 4)
	24c	Describe and explain any amendments to information provided at registration or in the protocol.	“Deviations between the protocol and the review” (page 13)
Support	25	Describe sources of financial or non-financial support for the review, and the role of the funders or sponsors in the review.	“Funding” (page 2)
Competing interests	26	Declare any competing interests of review authors.	“Competing interests” (page 14)
Availability of data, code and other materials	27	Report which of the following are publicly available and where they can be found: template data collection forms; data extracted from included studies; data used for all analyses; analytic code; any other materials used in the review.	“Data availability statement” (page 13)

From: Page MJ, McKenzie JE, Bossuyt PM, Boutron I, Hoffmann TC, Mulrow CD, et al. The PRISMA 2020 statement: an updated guideline for reporting systematic reviews. BMJ 2021;372:n71. doi: 10.1136/bmj.n71

For more information, visit: http://www.prisma-statement.org/

Text S2: Search strategy

Search strategies for
Human insulin vs short-acting insulin analogs for T1DM in children
(J Juul)
Updated searches performed 30 January 2026

**Total number of records identified:**	**16782 records**
**Number of duplicates excluded:**	**2629 records**
**Number of records in final list:**	**14153 records**
**Number of new records sent to authors:**	**8180 records**

Cochrane Central Register of Controlled Trials (2026, Issue 1) in the Cochrane Library (1645 hits)
#1 MeSH descriptor: [Insulin, Short-Acting] explode all trees
#2 MeSH descriptor: [Hypoglycemic Agents] explode all trees and with qualifier(s): [adverse effects - AE, therapeutic use - TU]
#3 (((fast* or rapid* or short*) NEAR/2 insulin*) or (insulin NEAR/2 (analog* or derivat* or prandial or lispro* or lyspro* or aspart* or glulisin* or glulysin*)) or novorapid or novolog or humalog or apidra or aspiara)
#4 #1 or #2 or #3
#5 MeSH descriptor: [Diabetes Mellitus, Type 1] explode all trees
#6 ((diabetes NEAR/1 ("type 1" or "type-1" or T1 or "insulin dependent" or insulin-dependent or juvenile or "sudden onset" or sudden-onset or autoimmune or auto-immune or brittle or ketosis-prone or ketosisprone or "ketosis prone")) or T1DM or IDDM)
#7 #5 or #6
#8 MeSH descriptor: [Adolescent] explode all trees
#9 MeSH descriptor: [Child] explode all trees
#10 MeSH descriptor: [Infant] explode all trees
#11 (baby or babies or newborn or neonat* or pediat* or paediat* or infant* or child* or boy* or girl* or toddler* or preschool* or pre-school* or school-child* or schoolchild* or teen* or youth* or adolescen* or juvenil* or student* or pubescen* or young* or minor*)
#12 #8 or #9 or #10 or #11
#13 #4 and #7 and #12

MEDLINE Ovid (1946 to 30 January 2026) (5522 hits)
1. exp Insulin, Short-Acting/
2. exp Hypoglycemic Agents/ae, tu, th [Adverse Effects, Therapeutic Use, Therapy]
3. (((fast* or rapid* or short*) adj2 insulin*) or (insulin adj2 (analog* or derivat* or prandial or lispro* or lyspro* or aspart* or glulisin* or glulysin*)) or novorapid or novolog or humalog or apidra or aspiara).mp. [mp=title, book title, abstract, original title, name of substance word, subject heading word, floating sub-heading word, keyword heading word, organism supplementary concept word, protocol supplementary concept word, rare disease supplementary concept word, unique identifier, synonyms, population supplementary concept word, anatomy supplementary concept word]
4. 1 or 2 or 3
5. exp Diabetes Mellitus, Type 1/
6. ((diabetes adj1 ("type 1" or "type-1" or T1 or "insulin dependent" or insulin-dependent or juvenile or "sudden onset" or sudden-onset or autoimmune or auto-immune or brittle or ketosis-prone or ketosisprone or "ketosis prone")) or T1DM or IDDM).mp. [mp=title, book title, abstract, original title, name of substance word, subject heading word, floating sub-heading word, keyword heading word, organism supplementary concept word, protocol supplementary concept word, rare disease supplementary concept word, unique identifier, synonyms, population supplementary concept word, anatomy supplementary concept word]
7. 5 or 6
8. exp adolescent/ or exp child/ or exp infant/
9. (baby or babies or newborn or neonat* or pediat* or paediat* or infant* or child* or boy* or girl* or toddler* or preschool* or pre-school* or school-child* or schoolchild* or teen* or youth* or adolescen* or juvenil* or student* or pubescen* or young* or minor*).mp. [mp=title, book title, abstract, original title, name of substance word, subject heading word, floating sub-heading word, keyword heading word, organism supplementary concept word,

protocol supplementary concept word, rare disease supplementary concept word, unique identifier, synonyms, population supplementary concept word, anatomy supplementary concept word]
10. 8 or 9
11. 4 and 7 and 10
12. exp randomized controlled trial/
13. (controlled clinical trial or retracted publication or retraction of publication).pt.
14. (randomized or placebo or randomly or trial or groups).ab.
15. drug therapy.fs.
16. 12 or 13 or 14 or 15
17. exp animals/ not humans.sh.
18. 16 not 17
19. 11 and 18

Embase Ovid (1974 to 30 January 2026) (6872 hits)
1. exp short acting insulin/
2. exp antidiabetic agent/ae, dt [Adverse Drug Reaction, Drug Therapy]
3. exp insulin derivative/ae, dt [Adverse Drug Reaction, Drug Therapy]
4. exp insulin lispro/
5. exp insulin aspart/
6. exp insulin glulisine/
7. (((fast* or rapid* or short*) adj2 insulin*) or (insulin adj2 (analog* or derivat* or prandial or lispro* or lyspro* or aspart* or glulisin* or glulysin*)) or novorapid or novolog or humalog or apidra or aspiara).mp. [mp=title, abstract, heading word, drug trade name, original title, device manufacturer, drug manufacturer, device trade name, keyword heading word, floating subheading word, candidate term word]
8. 1 or 2 or 3 or 4 or 5 or 6 or 7
9. exp insulin dependent diabetes mellitus/
10. ((diabetes adj1 ("type 1" or "type-1" or T1 or "insulin dependent" or insulin-dependent or juvenile or "sudden onset" or sudden-onset or autoimmune or auto-immune or brittle or ketosis-prone or ketosisprone or "ketosis prone")) or T1DM or IDDM).mp. [mp=title, abstract, heading word, drug trade name, original title, device manufacturer, drug manufacturer, device trade name, keyword heading word, floating subheading word, candidate term word]
11. 9 or 10
12. exp juvenile/
13. (baby or babies or newborn or neonat* or pediat* or paediat* or infant* or child* or boy* or girl* or toddler* or preschool* or pre-school* or school-child* or schoolchild* or teen* or youth* or adolescen* or juvenil* or student* or pubescen* or young* or minor*).mp. [mp=title, abstract, heading word, drug trade name, original title, device manufacturer, drug manufacturer, device trade name, keyword heading word, floating subheading word, candidate term word]
14. 12 or 13
15. 8 and 11 and 14
16. exp randomized controlled trial/ or controlled clinical trial/ or randomization/ or intermethod comparison/ or double blind procedure/ or human experiment/ or retracted article/
17. (random$ or placebo or compare or compared or comparison or (open adj label) or ((double or single or doubly or singly) adj (blind or blinded or blindly)) or parallel group$1 or crossover or cross over or ((assign$ or match or matched or allocation) adj5 (alternate or group$1 or intervention$1 or patient$1 or subject$1 or participant$1)) or assigned or allocated or (controlled adj7 (study or design or trial)) or volunteer or volunteers).ti,ab.
18. ((evaluated or evaluate or evaluating or assessed or assess) and (compare or compared or comparing or comparison)).ab.
19. trial.ti.
20. (erratum or tombstone).pt. or yes.ne.
21. 16 or 17 or 18 or 19 or 20
22. (random$ adj sampl$ adj7 ("cross section$" or questionnaire$1 or survey$ or database$1)).ti,ab. not (comparative study/ or controlled study/ or randomi?ed controlled.ti,ab. or randomly assigned.ti,ab.)
23. cross-sectional study/ not (exp randomized controlled trial/ or controlled clinical trial/ or controlled study/ or randomi?ed controlled.ti,ab. or control group$1.ti,ab.)
24. ((((case adj control$) and random$) not randomi?ed controlled) or (non random$ not random$) or "random field$" or (random cluster adj3 sample$)).ti,ab.
25. systematic review.ti,ab. not (trial or study).ti.

26. (review.ab. and review.pt.) not trial.ti.
27. "we searched".ab. and (review.ti. or review.pt.)
28. ("update review" or (databases adj4 searched)).ab.
29. (rat or rats or mouse or mice or swine or porcine or murine or sheep or lambs or pigs or piglets or rabbit or rabbits or cat or cats or dog or dogs or cattle or bovine or monkey or monkeys or trout or marmoset$1).ti. and animal experiment/
30. animal experiment/ not (human experiment/ or human/)
31. 22 or 23 or 24 or 25 or 26 or 27 or 28 or 29 or 30
32. 21 not 31
33. 15 and 32

LILACS (Bireme; 1982 to 30 January 2026) (822 hits)
(mh:(insulin, short-acting OR d06.472.699.587.200.400 OR d12.644.548.586.200.400 OR hypoglycemic agents OR d27.505.696.422 OR insulin lispro OR d06.472.699.587.200.400.500 OR d12.644.548.586.200.400.500 OR insulin aspart OR d06.472.699.587.200.400.100 OR d12.644.548.586.200.400.100)) OR ((((fast* OR rapid* OR short*) AND insulin*) OR (insulin AND (analog* OR derivat* OR prandial OR lispro* OR lyspro* OR aspart* OR glulisin* OR glulysin*)) OR novorapid OR novolog OR humalog OR apidra OR aspiara)) AND (mh:(diabetes mellitus, type 1 OR c18.452.394.750.124 OR c19.246.267 OR c20.111.327)) OR (((diabetes AND ("type 1" OR "type-1" OR t1 OR "insulin dependent" OR insulin-dependent OR juvenile OR "sudden onset" OR sudden-onset OR autoimmune OR auto-immune OR brittle OR ketosis-prone OR ketosisprone OR "ketosis prone")) OR t1dm OR iddm)) AND (mh:(adolescent OR m01.060.057 OR child OR m01.060.406 OR infant OR m01.060.703)) OR ((baby OR babies OR newborn OR neonat* OR pediat* OR paediat* OR infant* OR child* OR boy* OR girl* OR toddler* OR preschool* OR pre-school* OR school-child* OR schoolchild* OR teen* OR youth* OR adolescen* OR juvenil* OR student* OR pubescen* OR young* OR minor*)) AND (db:("LILACS"))

CINAHL (Ebsco host; 30 January 2026) (1012 hits)
S37 S13 AND S36
S36 S35 NOT S34
S35 S14 OR S15 OR S16 OR S17 OR S18 OR S19 OR S20 OR S21 OR S22 OR S23 OR S24 OR S25 OR S26 OR S27 OR S28
S34 S32 NOT S33
S33 MH (human)
S32 S29 OR S30 OR S31
S31 TI (animal model*)
S30 MH (animal studies)
S29 MH animals+
S28 AB (cluster W3 RCT)
S27 MH (crossover design) OR MH (comparative studies)
S26 AB (control W5 group)
S25 PT (randomized controlled trial)
S24 MH (placebos)
S23 MH (sample size) AND AB (assigned OR allocated OR control)
S22 TI (trial)
S21 AB (random*)
S20 TI (randomised OR randomized)
S19 MH cluster sample
S18 MH pretest-posttest design
S17 MH random assignment
S16 MH single-blind studies
S15 MH double-blind studies
S14 MH randomized controlled trials
S13 S4 AND S7 AND S12
S12 S8 OR S9 OR S10 OR S11
S11 TX (baby OR babies OR newborn OR neonat* OR pediat* OR paediat* OR infant* OR child* OR boy* OR girl* OR toddler* OR preschool* OR pre-school* OR school-child* OR schoolchild* OR teen* OR youth* OR adolescen* OR juvenil* OR student* OR pubescen* OR young* OR minor*)
S10 MH infant
S9 MH child

S8 MH adolescence
S7 S5 OR S6
S6 TX ((diabetes N1 (type 1 OR type-1 OR T1 OR insulin dependent OR insulin-dependent OR juvenile OR sudden onset OR sudden-onset OR autoimmune OR auto-immune OR brittle OR ketosis-prone OR ketosisprone OR ketosis prone)) OR T1DM OR IDDM)
S5 MH diabetes mellitus, type 1
S4 S1 OR S2 OR S3
S3 TX (((fast* OR rapid* OR short*) N2 insulin*) OR (insulin N2 (analog* OR derivat* OR prandial OR lispro* OR lyspro* OR aspart* OR glulisin* OR glulysin*)) OR novorapid OR novolog OR humalog OR apidra OR aspiara)
S2 MH Hypoglycemic Agents
S1 MH Insulin, Short-Acting

Science Citation Index EXPANDED (1900 to 30 January 2026) and Conference Proceedings Citation Index – Science (1990 to 30 January 2026) (Web of Science) (885 hits)
#4 #3 AND #2 AND #1
#3 TS=(baby or babies or newborn or neonat* or pediat* or paediat* or infant* or child* or boy* or girl* or toddler* or preschool* or pre-school* or school-child* or schoolchild* or teen* or youth* or adolescen* or juvenil* or student* or pubescen* or young* or minor*)
#2 TS=((diabetes NEAR/1 ("type 1" or "type-1" or T1 or "insulin dependent" or insulin-dependent or juvenile or "sudden onset" or sudden-onset or autoimmune or auto-immune or brittle or ketosis-prone or ketosisprone or "ketosis prone")) or T1DM or IDDM)
#1 TS=(((fast* or rapid* or short*) NEAR/2 insulin*) or (insulin NEAR/2 (analog* or derivat* or prandial or lispro* or lyspro* or aspart* or glulisin* or glulysin*)) or novorapid or novolog or humalog or apidra or aspiara)

The international HTA database (https://database.inahta.org/; 30 January 2026) (24 hits)
((baby OR babies OR newborn OR neonat* OR pediat* OR paediat* OR infant* OR child* OR boy* OR girl* OR toddler* OR preschool* OR pre-school* OR school-child* OR schoolchild* OR teen* OR youth* OR adolescen* OR juvenil* OR student* OR pubescen* OR young* OR minor*)) AND (((((diabetes AND ("type 1" OR "type-1" OR t1 OR "insulin dependent" OR insulin-dependent OR juvenile OR "sudden onset" OR sudden-onset OR autoimmune OR auto-immune OR brittle OR ketosis-prone OR ketosisprone OR "ketosis prone")) OR t1dm OR iddm)) OR ((diabetes mellitus, type 1)[mh])) AND ((((fast* OR rapid* OR short*) AND insulin*) OR (insulin AND (analog* OR derivat* OR prandial OR lispro* OR lyspro* OR aspart* OR glulisin* OR glulysin*)) OR novorapid OR novolog OR humalog OR apidra OR aspiara) OR (("Hypoglycemic Agents")[mh]) OR ((insulin, short-acting)[mh])))

Text S3: Supplementary results

Exploratory outcomes

All-cause mortality

One trial reported the outcome all-cause mortality [10]. The assessment time point was 26 weeks after the initiation of the intervention [10]. No participants died in either group (Fisher's exact test: p = 1.000) [10]. This outcome result was assessed at high risk of bias, and the certainty of the evidence was very low (**Table S2, Table S5**).

Postprandial glucose level

Six trials reported the outcome postprandial glucose level [1-4, 6, 9]. One additional trial assessed the outcome postprandial glucose level, but their data could not be included in the analyses (**Table S4**) [7]. The assessment time points ranged from three months to 16 weeks after the initiation of the intervention [1-4, 6, 9]. Fixed-effect meta-analysis showed a beneficial effect of rapid-acting insulin analogues (MD 0.63, 95% CI 0.20 to 1.06; I^2 = 60.0%; p = 0.0040; 6 trials). Random-effects meta-analysis showed no evidence of a difference (MD 0.57, 95% CI -0.38 to 1.52; I^2 = 60.0%; p = 0.2427; 6 trials) (**Fig. S18, Fig. S19**). Visual inspection of the forest plot and statistical tests (I^2 = 60.0%) indicated moderate heterogeneity. Trial Sequential Analysis confirmed the meta-analysis result (**Fig. S20**). This outcome result was assessed at high risk of bias, and the certainty of the evidence was very low (**Table S2, Table S5**).

Tests of interaction comparing the effects of age (p = 0.565), insulin lispro versus insulin aspart (p = 0.374), and method of delivery (p = 0.700) showed no evidence of differences (**Fig. S21, Fig. S22, Fig. S23**). The remaining predefined subgroup analyses could not be performed due to a lack of relevant data.

Continuous blood glucose monitoring

One trial reported the outcome continuous blood glucose monitoring [10]. The assessment time point was after 26 weeks after the initiation of the intervention [10]. The difference between maximum and minimum glucose levels measured over a 24 hour period was 4.0 mmol/L in the regular human insulin group and the rapid-acting insulin analogue group [10]. This outcome result was assessed at high risk (**Table S2**).

Nocturnal hypoglycemia

Four trials reported the outcome nocturnal hypoglycemia [1, 4, 5, 8]. Two additional trials assessed the outcome nocturnal hypoglycemia, but their data could not be included in the analyses (**Table S4**) [2, 6]. The assessment time points ranged from three months to 24 weeks after the initiation of the intervention [1, 4, 5, 8]. One trial reported the proportion of participants with nocturnal glycemia [8]. A total of 43/96 (44.8%) experienced nocturnal hypoglycemia in the regular human insulin group compared with 116/282 (41.1%) in the rapid-acting insulin analogue group (Fisher's exact test: p = 0.9042) [8]. This outcome result was assessed at high risk of bias, and the certainty of the evidence was very low (**Table S2, Table S5**). Three trials reported the rate of nocturnal hypoglycemia [1, 4, 5]. Fairchild 2000 reported a rate of 0.93 episodes per participant per three months when participants received regular human insulins (35 participants) and 1.03 episodes per participant per three months when participants received rapid-acting insulin analogues (35 participants) [1]. Holcombe 2002 reported a rate of 1.7 episodes per participant per one month when participants received regular human insulins (457 participants) and 1.0 episodes per participant per one month when participants received rapid-acting insulin analogues (457 participants) [4]. Ford-Adams 2003 reported a rate of 1 episodes per participant per four months when participants received regular human insulins (23

participants) and 1 episodes per participant per four months when participants received rapid-acting insulin analogues (23 participants) [5].

Analyses only including parallel trial results

Severe hypoglycemia

Two parallel trials reported the outcome severe hypoglycemia [8, 10]. The assessment time points ranged from 24 weeks to 26 weeks after randomization [8, 10]. A total of 9/108 (8.3%) experienced severe hypoglycemia in the regular human insulin groups compared with 20/282 (7.1%) in the rapid-acting insulin analogue groups [8, 10]. Meta-analysis showed no evidence of a difference (RR 1.21, 95% CI 0.59 to 2.48; $I^2 = 0.0\%$; p = 0.61; 2 trials) (**Fig. S24**). Trial Sequential Analysis showed that the meta-analysis was underpowered (no graph produced). This outcome result was assessed at high risk of bias (**Table S2**).

HbA1c

Three parallel trials reported the outcome HbA1c [5, 8, 10]. The assessment time points ranged from 20 weeks to 26 weeks after randomization [5, 8, 10]. Meta-analysis showed no evidence of a difference (MD 0.04, 95% CI -0.17 to 0.25; $I^2 = 0.0\%$; p = 0.71; 3 trials) (**Fig. S25**). Trial Sequential Analysis confirmed the meta-analysis result (**Fig. S26**). This outcome result was assessed at high risk of bias (**Table S2**).
