# Supplementary material for: Regular human insulins versus rapid-acting insulin analogues in children and adolescents with type 1 diabetes: a systematic review with meta-analysis and trial sequential analysis
Source: BMJ Open. 2026 May 14;16(5):e100186. doi: 10.1136/bmjopen-2025-100186 (PMC13182422; doi:10.1136/bmjopen-2025-100186)
Supplement: Supplementary file 2 [file bmjopen-2025-100186.reviewer_comments.pdf]

PEER REVIEW HISTORY

BMJ Open publishes all reviews undertaken for accepted manuscripts. Reviewers are asked to complete a checklist review form and are provided with free text boxes to elaborate on their assessment. These free text comments are reproduced below.

ARTICLE DETAILS

Title (Provisional)

Regular human insulins versus rapid-acting insulin analogues in children and adolescents with type 1 diabetes: a systematic review with meta-analysis and Trial Sequential Analysis

Authors

Petersen, Johanne Juul; Faltermeier, Pascal; Juul, Sophie; Kamp, Caroline Barkholt; Sillassen, Christina Dam Bjerregaard; Dos Santos, Tiago Jeronimo; Jakobsen, Janus C

VERSION 1 - REVIEW

**Reviewer**	**1**
**Name**	**Dewi, Ayu Putu Madri**
**Affiliation**	**Amsterdam UMC Location AMC, Epidemiology and Data Science**
**Date**	**12-Mar-2025**
**COI**	**None**

I am an Open Science researcher, who has checked your manuscript for its adherence to a number of Open Science practices.

Below you find the checklist that I used to assess your manuscript for adherence to a number of these practices.

Based on this assessment, I would suggest the following refinements/additions:

1. *Availability of a protocol: OK (citation and in supplementary materials).*
2. *Pre-registration: OK (PROSPERO).*
3. *Open Materials: N/A.*
4. *Open Data: Authors state "The authors confirm that the data supporting the findings of this study are available within the article and its supplementary materials". That is probably true for a meta-analysis like this. OK.*
5. *Open Code: Please provide (a link to) the code used for the analyses in this study.*
6. *Citations: Please provide citations for your codes.*
7. *Software and tools used are described: Please explicitly mention which software and tools were used in your study, including references to versions.*
8. *Open access publication: OK.*
9. *Reporting checklist provided: OK.*
10. *Preprint: Please state explicitly whether and where the preprint of this study can be found.*

**Reviewer**	**2**
**Name**	**Kelson, Zoe**
**Affiliation**	**University of Exeter, Mathematics**
**Date**	**15-May-2025**
**COI**	**None**

This systematic review of randomised clinical trials with meta-analysis and Trial Sequential Analysis aims to assess the beneficial and harmful effects of regular human insulins versus rapid acting insulin analogues in children and adolescents with type 1 diabetes.

Reviewer comments:

"Systematic review registration: PROSPERO ID: CRD42024508625"

and

"The updated methodology was conducted according to our protocol, registered in the PROSPERO database (ID: CRD42024508625) before our literature searches. "

The authors have suitably registered the study protocol. Thanks for providing a copy.

"This systematic review is reported based on the Preferred Reporting Items for Systematic Reviews and Meta-Analysis (PRISMA) guidelines (Text S1)."

Thanks for providing a copy of the PRISMA checklist.

Can the Abstract please provide a summary of the Methods?

"The certainty of evidence was very low mainly due to high risk of bias and small sample sizes. Meta-analysis showed no evidence of a difference between regular human insulins and rapid-acting insulin analogues on severe hypoglycaemia (risk ratio (RR) 1.28, 95% confidence interval (CI) 0.81 to 2.03; I 2 = 0.0%; p = 0.2851; 9 trials), ketoacidosis (RR 0.88, 95% CI 0.26 to 2.93; I 2 = 0.0%; p = 0.8593; 2 trials), and serious adverse events (RR 1.00, 95% CI 0.44 to 2.25; I 2 = 0.0%; p = 0.9958; 2 trials). Trial Sequential Analysis showed that all meta-analyses of primary outcomes were underpowered." [Abstract]

The study findings have been clearly and concisely communicated in the Abstract.

"Randomised clinical trials irrespective of publication year, status, and language were included. "

The authors have done well to keep the review open.

"All databases were searched from their inception until February 6, 2024"

Can the review please be updated prior to publication?

"Three authors (JJP, PF, and CBS) independently screened relevant trials. Three authors (JJP, SJ and PF) independently (working in pairs) extracted data using a standardised data extraction sheet and assessed the risks of bias based on the Cochrane Risk of Bias tool, version 2 (RoB 2).18,19 Discrepancies were resolved through discussion, or, if required, through discussion with a third author (JCJ)."

The authors have followed rigorous article sceening, selection and data extraction processes, and have satisfactorily assessed study quality.

" Several subgroup analyses were planned."

Can the planned subgroup analyses please be specified (i.e. listed) here in the methods?

"Both random-effects (inverse variance and DerSimonian-Laird) and fixedeffect (Mantel-Haenszel) meta-analyses were performed for all analyses, and we primarily reported the most conservative results (highest p-value). The least conservative results were considered sensitivity analyses"

Appropriate modelling methods have been applied.

Can the authors please clarify how they assessed statistical heterogeneity in the methods?

"Trial Sequential Analysis was used to control for random errors.24-32 Grading Recommendations Assessment Development Evaluation (GRADE) was used to assess the certainty of evidence for all outcomes."

The authors have rigorously assessed and reported on the certainty of evidence.

Can the authors please assess the risk of publication bias (e.g. applying funnel plots and conducting statistical tests for study study effect, such as Egger's or Begg's tests)?

Can the authors please conduct a leave-one-out sensitivity analysis to help demonstrate the robustness of the study findings to any one article?

The study outcomes have been reported accurately and completely in the Results section.

"The results of this review may be susceptible to publication bias, because only a limited amount of grey literature was identified. • Since the review is based on data from cross-over trials, it is not possible to draw conclusions about long-term effects, and the relevance of short-term effects may be limited due to the potential risk of carry-over effects" [strengths and limitations]

and

"This systematic review also has limitations..."

A thorough discussion on the study limitations has been provided.

**Reviewer**	**3**
**Name**	**Eledrisi, Mohsen Saleh**
**Affiliation**	**Hamad Medical Corporation, Medicine**
**Date**	**13-Jun-2025**
**COI**	**None**

This is an interesting metaanalysis and systematic review about an important clinical issue. The following are noted.

• Originality – The work is original with no similar published data in the literature. It adds an importance piece to the existing evidence about the subject.

• Importance of work to readers – the topic is important to health care professionals dealing with persons with type 1 diabetes as well as patients and policymakers.

• Evidence-based medicine – the paper is presented in a scientific and reliable format.

• Research question - is clearly defined and appropriately answered.

• Study design – is appropriate and adequate.

• Participants - are adequately described and well defined.

• Methods - are adequately described. The use of methods is appropriate including metaanalysis, systematic review and sequential . There are no ethical concerns.

• Results – are clearly stated and appear credible.

• Interpretation and conclusions - are clear and supported by the presented data. The strengths and limitations of the paper are clearly stated.

• References - are fair. I suggest adding published metaanalysis on the comparison of meal insulins (human versus analoges) in adults with type 1 diabetes.

• Abstract/summary/key messages – are clear and accurately reflect the puropse of the paper.

• Reporting standards – the paper complies with standards of metaanalysis and systematic reviews.

- Please clarify the point on side effects. It is mentioned that 1 trial reported “non-serious adverse events “ and 5 trials reported “outcome adverse events”. What are the nature of these side effects?and was there a difference between these 2 groups?

**Reviewer**	**4**
**Name**	**Tung, Tao-Hsin**
**Affiliation**	**Taizhou Hospital of Zhejiang Province**
**Date**	**11-Jan-2026**
**COI**	**None**

This article addresses an interesting research topic; however, several key aspects require further elaboration and supplementation to enhance its rigor and completeness, as detailed below:

1. Within the Introduction section, the authors are advised to supplement a summary of relevant previous systematic reviews. Specifically, this should include a clear delineation of the limitations of these existing reviews, as well as an explicit justification for conducting the present systematic review (i.e., the research rationale).

2. The search date reported in the manuscript should be updated. To ensure the currency and comprehensiveness of the systematic review, it is recommended that the search be re-run and the updated search date be clearly specified.

3. It is recommended that the kappa statistics for inter-reviewer agreement during both the study selection and data extraction phases be reported.

4. The authors are required to include a comprehensive discussion of the heterogeneity observed among the included studies, including potential sources of heterogeneity and the implications of this heterogeneity for the interpretation of the review results.

5. A critical appraisal of all included trials should be added to the manuscript. This appraisal should evaluate the methodological quality and risk of bias of each included study, which is essential for assessing the reliability of the aggregated results.

6. The authors should provide readers with specific details regarding the clinical applicability of the study's findings. This includes clarifying how the review results can be translated into practical clinical decision-making and applied in real-world clinical settings.

7. The Discussion section should explicitly address how the present review advances or updates the existing body of evidence. This requires a clear comparison with previous relevant studies/reviews to highlight the novel contributions and added value of the current work.

VERSION 1 - AUTHOR RESPONSE

Reviewer 1

I am an Open Science researcher, who has checked your manuscript for its adherence to a number of Open Science practices. Below you find the checklist that I used to assess your manuscript for adherence to a number of these practices. Based on this assessment, I would suggest the following refinements/additions.

Author response: We thank the reviewer for this comment.

1. Availability of a protocol: OK (citation and in supplementary materials).

Author response: We thank the reviewer for this comment.

2. Pre-registration: OK (PROSPERO).

Author response: We thank the reviewer for this comment.

3. Open Materials: N/A.

Author response: NA.

4. Open Data: Authors state "The authors confirm that the data supporting the findings of this study are available within the article and its supplementary materials". That is probably true for a meta-analysis like this. OK.

Author response: We thank the reviewer for this comment.

5. Open Code: Please provide (a link to) the code used for the analyses in this study.

Author response: Analyses were performed using Stata version 17 (StataCorp LLC, College Station, TX, USA) using the syntax 'meta'. This has now been clarified including a link.

6. Citations: Please provide citations for your codes.

Author response: We have now provided citations for our code.

7. Software and tools used are described: Please explicitly mention which software and tools were used in your study, including references to versions.

Author response: We have now included the names, versions, and references for software used in the study.

8. Open access publication: OK.

Author response: We thank the reviewer for this comment.

9. Reporting checklist provided: OK.

Author response: We thank the reviewer for this comment.

10. Preprint: Please state explicitly whether and where the preprint of this study can be found.

Author response: No preprints of this study were published.

Reviewer 2

This systematic review of randomised clinical trials with meta-analysis and Trial Sequential Analysis aims to assess the beneficial and harmful effects of regular human insulins versus rapid acting insulin analogues in children and adolescents with type 1 diabetes.

1. "Systematic review registration: PROSPERO ID: CRD42024508625" and "The updated methodology was conducted according to our protocol, registered in the PROSPERO database (ID: CRD42024508625) before our literature searches. " The authors have suitably registered the study protocol. Thanks for providing a copy.

Author response: We thank the reviewer for this comment.

2. "This systematic review is reported based on the Preferred Reporting Items for Systematic Reviews and Meta-Analysis (PRISMA) guidelines (Text S1)." Thanks for providing a copy of the PRISMA checklist.

Author response: We thank the reviewer for this comment.

3. Can the Abstract please provide a summary of the Methods?

Author response: We have now included more information on the methods in the Abstract.

4. "The certainty of evidence was very low mainly due to high risk of bias and small sample sizes. Meta-analysis showed no evidence of a difference between regular human insulins and rapid-acting insulin analogues on severe hypoglycaemia (risk ratio (RR) 1.28, 95% confidence interval (CI) 0.81 to 2.03; $I^2 = 0.0\%$; $p = 0.2851$; 9 trials), ketoacidosis (RR 0.88, 95% CI 0.26 to 2.93; $I^2 = 0.0\%$; $p = 0.8593$; 2 trials), and serious adverse events (RR 1.00, 95% CI 0.44 to 2.25; $I^2 = 0.0\%$; $p = 0.9958$; 2 trials). Trial Sequential Analysis showed that all meta-analyses of primary outcomes were underpowered." [Abstract]. The study findings have been clearly and concisely communicated in the Abstract.

Author response: We thank the reviewer for this comment.

5. "Randomised clinical trials irrespective of publication year, status, and language were included. " The authors have done well to keep the review open.

Author response: We thank the reviewer for this comment.

6. "All databases were searched from their inception until February 6, 2024". Can the review please be updated prior to publication?

Author response: We have now updated the search on January 30, 2026.

7. "Three authors (JJP, PF, and CBS) independently screened relevant trials. Three authors (JJP, SJ and PF) independently (working in pairs) extracted data using a standardised data extraction sheet and assessed the risks of bias based on the Cochrane Risk of Bias tool, version 2 (RoB 2).18,19 Discrepancies were resolved through discussion, or, if required, through discussion with a third author (JCJ)." The authors have followed rigorous article sceening, selection and data extraction processes, and have satisfactorily assessed study quality.

Author response: We thank the reviewer for this comment.

8. " Several subgroup analyses were planned." Can the planned subgroup analyses please be specified (i.e. listed) here in the methods?

Author response: We have now included the preplanned subgroup analyses in the systematic review.

9. "Both random-effects (inverse variance and DerSimonian-Laird) and fixedeffect (Mantel-Haenszel) meta-analyses were performed for all analyses, and we primarily reported the most conservative results (highest p-value). The least conservative results were considered sensitivity analyses". Appropriate modelling methods have been applied.

Author response: We thank the reviewer for this comment.

10. Can the authors please clarify how they assessed statistical heterogeneity in the methods?

Author response: Assessment of statistical heterogeneity was originally described in our protocol. We have now added a detailed description of the methods used to assess heterogeneity in the Methods section of the review.

11. "Trial Sequential Analysis was used to control for random errors.24-32 Grading Recommendations Assessment Development Evaluation (GRADE) was used to assess the certainty of evidence for all outcomes." The authors have rigorously assessed and reported on the certainty of evidence.

Author response: We thank the reviewer for this comment.

12. Can the authors please assess the risk of publication bias (e.g. applying funnel plots and conducting statistical tests for study study effect, such as Egger's or Begg's tests)?

Author response: We specified the following in our protocol regarding assessment of publication

bias: "*We will use a funnel plot to assess reporting bias if ten or more trials are included. We will visually inspect funnel plots to assess the risk of bias. We are aware of the limitations of a funnel plot (i.e., a funnel plot assesses small study bias). From this information, we will assess possible reporting bias. For dichotomous outcomes, we will test asymmetry with the Harbord test if $\tau 2$ is less than 0.1 and with the Rücker test if $\tau 2$ is more than 0.1.*". Since we did not include ten trials in any meta-analysis, no statistical assessment of reporting bias was conducted. This has now been clarified in the Results section.

13. Can the authors please conduct a leave-one-out sensitivity analysis to help demonstrate the robustness of the study findings to any one article?

Author response: We respectfully disagree with the reviewer on the relevance of a leave-one-out sensitivity analysis, as we do not believe this sensitivity analysis would meaningfully strengthen the review. The pooled estimates are already highly uncertain due to the small number of trials and limited sample sizes, and any changes would likely reflect this imprecision rather than true study influence. Moreover, our assessment of heterogeneity did not identify clear signals of inconsistency or outlier effects. We therefore consider such an analysis unlikely to alter the interpretation of the findings.

14. The study outcomes have been reported accurately and completely in the Results section.

Author response: We thank the reviewer for this comment.

15. "The results of this review may be susceptible to publication bias, because only a limited amount of grey literature was identified. • Since the review is based on data from cross-over trials, it is not possible to draw conclusions about long-term effects, and the relevance of short-term effects may be limited due to the potential risk of carry-over effects" [strengths and limitations] and "This systematic review also has limitations...". A thorough discussion on the study limitations has been provided.

Author response: We thank the reviewer for this comment.

Reviewer 3

1. This is an interesting metaanalysis and systematic review about an important clinical issue. The following are noted.

Author response: We thank the reviewer for this comment.

2. Originality – The work is original with no similar published data in the literature. It adds an importance piece to the existing evidence about the subject.

Author response: We thank the reviewer for this comment.

3. Importance of work to readers – the topic is important to health care professionals dealing with persons with type 1 diabetes as well as patients and policymakers.

Author response: We thank the reviewer for this comment.

4. Evidence-based medicine – the paper is presented in a scientific and reliable format.

Author response: We thank the reviewer for this comment.

5. Research question - is clearly defined and appropriately answered.

Author response: We thank the reviewer for this comment.

6. Study design – is appropriate and adequate.

Author response: We thank the reviewer for this comment.

7. Participants - are adequately described and well defined.

Author response: We thank the reviewer for this comment.

8. Methods - are adequately described. The use of methods is appropriate including metaanalysis, systematic review and sequential. There are no ethical concerns.

Author response: We thank the reviewer for this comment.

9. Results – are clearly stated and appear credible.

Author response: We thank the reviewer for this comment.

10. Interpretation and conclusions - are clear and supported by the presented data. The strengths and limitations of the paper are clearly stated.

Author response: We thank the reviewer for this comment.

11. References - are fair. I suggest adding published metaanalysis on the comparison of meal insulins (human versus analoges) in adults with type 1 diabetes.

Author response: We have now included a reference for a meta-analysis on the comparison of rapid-acting insulin analogues with regular human insulin in adults with type 1 diabetes.

12. Abstract/summary/key messages – are clear and accurately reflect the puropse of the paper.

Author response: We thank the reviewer for this comment.

13. Reporting standards – the paper complies with standards of metaanalysis and systematic reviews.

Author response: We thank the reviewer for this comment.

14. Please clarify the point on side effects. It is mentioned that 1 trial reported “non-serious adverse events “ and 5 trials reported “outcome adverse events”. What are the nature of these side effects?and was there a difference between these 2 groups?

Author response: We thank the reviewer for this comment. One trial reported non-serious adverse events separately for the intervention and control groups. Five crossover trials reported adverse events without specifying whether they were serious or non-serious, and the data were not reported in a way suitable for meta-analysis (e.g., it was unclear in which group the events occurred). Therefore, these data have been summarised descriptively in Table S4. This clarification has now been added to the manuscript.

Reviewer 4

1. This article addresses an interesting research topic; however, several key aspects require further elaboration and supplementation to enhance its rigor and completeness, as detailed below.

Author response: We thank the reviewer for this comment.

1. Within the Introduction section, the authors are advised to supplement a summary of relevant previous systematic reviews. Specifically, this should include a clear delineation of the limitations of these existing reviews, as well as an explicit justification for conducting the present systematic review (i.e., the research rationale).

Author response: We thank the reviewer for this suggestion. While our protocol included an overview of previous systematic reviews, we have now updated the Introduction section to provide a summary of relevant previous reviews, highlight their limitations, and clearly justify the rationale for conducting the present systematic review.

2. The search date reported in the manuscript should be updated. To ensure the currency and comprehensiveness of the systematic review, it is recommended that the search be re-run and the updated search date be clearly specified.

Author response: We have now updated the search on January 30, 2026.

3. It is recommended that the kappa statistics for inter-reviewer agreement during both the study selection and data extraction phases be reported.

Author response: We thank the reviewer for this suggestion. While kappa statistics can quantify inter-reviewer agreement, the Cochrane Handbook does not recommend their routine use. Our review employed a structured process with independent screening and data extraction by two reviewers, in line with best-practice methods to ensure reliability.
Therefore, we believe that the procedures we used sufficiently ensure the validity of study selection and data extraction, even without reporting kappa statistics.

4. The authors are required to include a comprehensive discussion of the heterogeneity observed among the included studies, including potential sources of heterogeneity and the implications of this heterogeneity for the interpretation of the review results.

Author response: We have now included the following in the Discussion: "*Lastly, the included trials differed in population, intervention, timing of intervention, and definition and measurement of outcomes. This clinical heterogeneity could potentially limit the interpretability of pooled results. However, several subgroup and sensitivity analyses were conducted, and no clear evidence of statistical heterogeneity was identified.*"

5. A critical appraisal of all included trials should be added to the manuscript. This appraisal should evaluate the methodological quality and risk of bias of each included study, which is essential for assessing the reliability of the aggregated results.

Author response: We thank the reviewer for this suggestion. Risk of bias was assessed for all included trials, and these assessments are presented in Table S2. Additionally, we evaluated risk of bias for all outcomes, which is included in the Summary of Findings table (Table S5). This has now been clarified in the Methods section of the review.

6. The authors should provide readers with specific details regarding the clinical applicability of the study's findings. This includes clarifying how the review results can be translated into

practical clinical decision-making and applied in real-world clinical settings.

Author response: We have now included the following in the Discussion: "*This systematic review found no evidence of a difference between regular human insulin and rapid-acting insulin analogues on clinical outcomes. In practice, given the likely similar effects on outcomes such as HbA1c and hypoglycaemia, other factors, including cost, availability, and patient or caregiver preferences, may guide clinical decision-making*."

7. The Discussion section should explicitly address how the present review advances or updates the existing body of evidence. This requires a clear comparison with previous relevant studies/reviews to highlight the novel contributions and added value of the current work.

Author response: We have now included a section in the Discussion addressing how the present review compares to previous reviews.
